# DNA methylation regulator-mediated modification pattern defines tumor microenvironment immune infiltration landscape in colon cancer

**DOI:** 10.3389/fgene.2022.1008644

**Published:** 2022-10-06

**Authors:** Shijin Yuan, Yuzhen Gao, Yan Xia, Zhuo Wang, Xian Wang

**Affiliations:** ^1^ Department of Medical Oncology, Cancer Institute of Zhejiang University, Sir Run Run Shaw Hospital, School of Medicine, Zhejiang University, Hangzhou, China; ^2^ Department of Clinical Laboratory, Sir Run Run Shaw Hospital, School of Medicine, Zhejiang University, Hangzhou, China

**Keywords:** DNA methylation, immunotherapy, tumor microenvironment, colon cancer, biomarker

## Abstract

Emerging evidence implies a non-negligible role of DNA methylation in tumor immunity, however, its comprehensive impact on tumor microenvironment (TME) formation and immune activation remains unclear. In this study, we integrated 24 DNA methylation regulators among 754 colon cancer patients to distinguish different modification patterns *via* an unsupervised clustering method, and explore their TME immune characteristics. Three DNA methylation modification patterns with distinct prognosis and biological behaviors were identified, consistent with three known phenotypes of immune-inflamed, immune-excluded, and immune-desert. We then determined a DNA methylation gene signature and constructed a DNA methylation score (DMS) to quantify modification patterns individually through principal component analysis algorithms. DMS-low group had characteristics of specific molecular subtypes, including microsatellite instability, CpG island methylator phenotype positive, and mutant BRAF, presented by increased mutation burden, activation of DNA damage repair and immune-related pathways, highly TME immune cells infiltration, and hence, a preferable prognosis. Further, low DMS was also demonstrated to be correlated to better response and prolonged survival of anti-PD-L1 antibody, indicating that DMS could be considered as an effective predictive tool for immunotherapy. In conclusion, our work presented a landscape of different DNA methylation modification patterns, and their vital role in the formation of TME diversity and complexity, which could help to enhance understanding of TME immune infiltration characteristics and more importantly, guide immunotherapy strategies more effectively and personalized.

## Introduction

Colon cancer is common worldwide and remains one of the leading causes of cancer-related mortality (Sung et al., 2021). As a biologically heterogeneous disease, colon cancer derives from the accumulation of a series of genetic and epigenetic changes that transform normal glandular epithelium into malignant invasive adenocarcinoma. Now, it is appreciated that there are multiple molecular pathways involved in these genetic mutations and epigenetic alterations during colon cancer development, including microsatellite instability (MSI), the CpG island methylator phenotype (CIMP), chromosome instability (CIN), and somatic mutations of critical oncogenes like BRAF and KRAS (Nguyen et al., 2020).

Epigenetics, referring to heritable alterations in gene expression that are not dependent on changes in the DNA sequence, plays an important role in the pathogenesis of colon cancer (Lao and Grady. 2011). Therein, aberrant DNA methylation, one of the most widely studied epigenetic modifications, could lead to the dysregulation of gene expression in colon cancer. DNA methylation is a reversible modification process mediated by DNA methyltransferases (DNMTs) that facilitate the catalytic addition of methyl groups to the fifth position of the cytosine of CpG dinucleotides to generate 5-methylcytosine (5mC). On the contrary, 5mC could be reversed to unmodified cytosine through TET dioxygenase-mediated oxidation (Bestor 2000). Over the past two decades, emerging studies have elucidated the epigenetically regulatory mechanism of DNA methylation in colon cancer-specific gene expression patterns. Hypermethylation in the promoter region could silence the expression of tumor-suppressor genes, and contribute to loss of function. For instance, MSI, one of the hallmarks of molecular subtypes of colon cancer (Dienstmann et al., 2017), is the consequence of a deficiency in the DNA mismatch repair (MMR) system, which is not only due to the genetic mutation of MMR-related genes, but also the results of hypermethylation of MLH1 gene (Herman et al., 1998). On the contrary, global hypomethylation of the genome has been demonstrated to influence colon cancer development through inducing CIN and global loss of imprinting (Suter et al., 2004). These insights have improved our understanding of colon cancer pathophysiology and provide clues to discover novel biomarkers and therapeutic targets.

Recently, immunotherapy, especially the inhibitor targeting immune checkpoints like CTLA-4, PD-1, or PD-L1, has achieved durable anti-tumor activity in a range of cancer types. However, there are many patients, particularly in colon cancer with microsatellite stable (MSS), do not benefit from this advanced treatment (Le et al., 2015). The major reason is thought to lack lymphocytes infiltration in the MSS tumor microenvironment (TME), forming a “immune-desert” phenotype and resulting a weak immunoreactivity to the immunotherapy (Chen and Mellman, 2017). Increasing evidence demonstrated DNA methylation regulators mediated regulation is critical in anti-tumor immune response through involving in many processes of the cancer-immunity cycle (Cao and Yan, 2020). To be specific, DNA methylation-associated mutagenesis could generate tumor neoantigens (Alexandrov et al., 2013). In tumor cells, DNMTs could suppress the expression of MHC-I to dysregulate the antigen-presenting machinery, and suppress the expression of pro-inflammatory chemokines, such as CXCL9 and CXCL10, which are required by effector T-cells to permeate the TME and execute an immune attack (Chen et al., 2017; Luo et al., 2018). Hypermethylation of the PD-L1 promotor region inhibits its expression and leads to an inferior prognosis in various cancer types (Goltz et al., 2017; Heiland et al., 2017). Meanwhile, in immune cells, MBD2, the “reader” of methylated DNA, is necessary to induce dendritic cells phenotypic activation and then initiate the T cell response (Cook et al., 2015). By contrast, TET2 and HDAC2, the “eraser” of DNA methylation and histone acetylation respectively, coordinate to suppress IL-6 expression of dendritic cells, inhibiting the inflammatory response (Zhang et al., 2015).

However, to date, the majority of studies focus on the function of one or two DNA methylation regulators, which cannot reflect the whole landscape of DNA methylation in the formation of tumor-permissive immune environment. Therefore, comprehensive recognition of the TME immune characteristics mediated by multiple DNA methylation regulators, including TME infiltrating immune cells and activity of immune/inflammatory-related pathways, could enhance our understanding of TME immune regulation, and further provide novel perspectives for cancer immunotherapy. In this study, we integrated the transcriptomic and clinical information of 754 colon cancer samples to identify DNA methylation modification patterns with distinct TME immune characteristics, which were highly consistent with three known immune phenotypes, including immune-inflamed, immune-excluded, and immune-desert phenotype, respectively. In addition, we determined the DNA methylation gene signature and constructed a scoring system to quantify modification patterns for individual patients, which could be served as an effective biomarker for predicting the efficacy and prognosis of immunotherapy.

## Materials and methods

### Study design, colon cancer datasets collection, and data processing

Supplementary Figure S1A depicted the workflow of our present study. Generally, we searched publicly available transcriptomic data and clinical annotation of colon cancer samples from the Gene-Expression Omnibus (GEO) and the Cancer Genome Atlas (TCGA) database. Seven eligible colon cancer datasets with comprehensive survival information [GSE39582 (*n* = 562), GSE38832 (*n* = 122), GSE39084 (*n* = 70), GSE72970 (*n* = 124), GSE103479 (*n* = 155), GSE87211 (*n* = 196), and TCGA-colon adenocarcinoma cohort (COAD, *n* = 430)], including 1,659 patients, were collected for our further analysis (Supplementary Table S1). For the RNA sequencing data of the TCGA-COAD cohort, the fragments per kilobase of transcript per million mapped reads (FPKM) value of each sample was downloaded from the Genomic Data Commons (GDC, https://portal.gdc.cancer.gov/) by the “*TCGAbiolinks*” R package directly (Colaprico et al., 2016). And for the microarray data of the GSE cohort, we used the normalized matrix files downloaded from GEO. The somatic mutation data, copy number variation (CNV) data, and TCGA pan-cancer RNA sequencing data were acquired from the University of California Santa Cruz (UCSC) Xena browser (https://xenabrowser.net).

Moreover, we also included an immunotherapeutic cohort (IMvigor210, *n* = 348), urothelial carcinoma treated with anti-PD-L1 antibody atezolizumab, to evaluate the effect of DNA methylation modification in immunotherapy. The expression data and clinical information were available from the “*IMvigor210*” R package (Mariathasan et al., 2018).

### Unsupervised clustering for 24 DNA methylation regulators

After a systematic review of published articles, we identified a total of 24 DNA methylation regulators, including 3 writers (DNMT1, DNMT3A, DNMT3B), 3 erasers (TET1, TET2, TET3), and 18 readers (MBD1, MBD2, MBD3, MBD4, ZBTB33, ZBTB38, ZBTB4, ZBTB24, UHRF1, UHRF2, MECP2, UNG, TDG, NTHL1, SMUG1, NSUN2, MGMT, DMAP1). The protein-protein interactions (PPI) network among 24 regulators were analyzed by the STRING interaction database (https://string-db.org/) (Szklarczyk et al., 2019) and visualized by the Cytoscape software (Shannon et al., 2003).

Three GEO datasets (GSE39582, GSE38832, and GSE39084) with the same microarray platform and no prognostic differences were integrated as meta-cohort (*n* = 754) to identify different DNA methylation modification patterns mediated by 24 regulators (Supplementary Figure S2A). The non-biological technical biases caused batch effect among each cohort was eliminated by the “*ComBat*” algorithm of the “*sva*” R package. Based on the expression of 24 regulators, the Nonnegative Matrix Factorization (NMF) method was used to determine different DNA methylation modification patterns through the “*NMF*” R package (Gaujoux and Seoighe. 2010).

### Identification of differentially expressed genes and generation of DNA methylation gene signature

First, to identify DNA methylation phenotype-related genes, we used the empirical Bayesian approach of the “*limma*” R package to determine differentially expressed genes (DEGs) among three DNA methylation modification patterns (Ritchie et al., 2015). The adjusted *p*-value was set as < 0.01 to select significant DEGs. Then, we performed a univariate Cox regression analysis to confirm the DEGs that were significantly related to the prognosis (*p*-value < 0.01). Subsequently, based on the expression of these prognostic DEGs, we conducted the second NMF clustering algorithm to obtain DNA methylation gene clusters as well as validate their stability. Furthermore, through the principal component analysis (PCA) method, we used these prognostic DEGs to construct the DNA methylation gene signature, termed DNA methylation score (DMS), which could quantify the DNA methylation modification pattern for each patient. The procedure of establishing the DMS was similar to a previous study, and we added the principal component 1 and 2 to acquire the signature scores (Zhang et al., 2020; Gao et al., 2021). The formula of DMS was expressed as follow:

$$DMS=\Sigma \;\left(PC1_{i}+PC2_{i}\right)$$

where *i* is the expression of DNA methylation signature genes.

### Gene set variation analysis, gene set enrichment analysis, and functional annotation

To investigate the difference in the biological processes among different DNA methylation modification patterns, gene clusters, and DMS groups, we performed gene set variation analysis (GSVA) and gene set enrichment analysis (GSEA) analyses through the “*GSVA*” and “*clusterProfiler*” R packages, respectively (Yu et al., 2012; Hanzelmann et al., 2013). The hallmark gene sets (h.all.v7.5.1.symbols) and the Kyoto Encyclopedia of Genes and Genomes (KEGG) gene sets (c2.cp.kegg.v7.5.1.symbols) were downloaded from the MSigDB database for running enrichment analysis (http://www.gsea-msigdb.org/gsea/msigdb/). All enrichment *p*-values were adjusted by the Benjamini-Hochberg methods and less than 0.05 were considered statistically significant (Thissen et al., 2002). Moreover, we performed Gene Ontology (GO) functional annotation for DEGs *via* the “*clusterProfiler*” R package, with the cutoff value of false discovery rate (FDR) < 0.05.

We additionally collected 18 classical biological processes constructed by Mariathasan et al. (2018), including: 1) CD8 T effector; 2) DNA damage repair (DDR); 3) antigen-processing machinery (APM); 4) immune checkpoint; 5) cell cycle regulators; 6) Fanconi anemia; 7) pan-fibroblast TGFβ response signature (Pan-F-TBRS); 8) epithelial-mesenchymal transition (EMT) markers including EMT1, EMT2 and EMT3; 9) WNT targets; 10) fibroblast growth factor receptor 3 (FGFR3) related signature; 11) cell cycle signature; 12) mismatch repair 13) homologous recombination; 14) nucleotide excision repair; 15) DNA replication; 16) base excision repair. The detailed gene set of the corresponding pathway was provided in Supplementary Table S2.

### Estimation of the tumor microenvironment infiltrating cell abundance and the immune infiltration score

We used three different algorithms, including the single-sample gene-set enrichment analysis (ssGSEA) algorithm of the “*GSVA*” R package, the CIBERSORT method, and the Tumor Immune Estimation Resource (TIMER) database, to evaluate the infiltrating abundance of various TME immune cells, such as B cell, CD8+ T cell, dendritic cell, and macrophage et al. The gene sets of each type TME infiltrating cell were extracted from the study of Charoentong and listed in Supplementary Table S3 (Charoentong et al., 2017).

Besides, through applying “*xCell*” and “*ESTIMATE*” methods, we calculated the TME stromal score, immune score, estimate score, and microenvironment score based on signature gene expression to infer the fraction of stromal and immune cells in colon cancer samples. The calculation was performed by the “*xCell*” and “*estimate*” R packages, respectively (Yoshihara et al., 2013; Aran et al., 2017).

### Cell culture

The colorectal cancer cell line HCT116 and normal colonic epithelial cell line NCM460 were purchased from Type Culture Collection of the Chinese Academy of Science (Shanghai, China). HCT116 cells were cultured in McCoy’s 5A Medium (16600-082, Gibco) with 10% fetal bovine serum (10099141C, Gibco) in 5% CO_2_ at 37°C. NCM460 cells were cultured in RPMI 1640 Medium (118575-093, Invitrogen) with same condition.

### RNA extraction and real-time quantitative reverse transcription-polymerase chain reaction)

Total RNA was isolated using TRIzol™ Reagent (15596026, Invitrogen) and quantified with a NanoDrop 2000™ (Thermo Fisher Scientific, United States). 1 μg RNA was used for the reverse transcription reaction to generate cDNA through the PrimeScript™ RT Reagent Kit with gDNA Eraser (RR047A, TaKaRa) according to the manufacturer’s protocols. The mRNA expression was determined by rt-qPCR, which was performed using Ultra SYBR Mixture (CW0957M, CWBIO) and a LightCycler^®^ 480 II system (Roche, Shanghai, China). The mRNA expression of ACTB was used as a reference. The primers used in this study were listed in Supplementary Table S4.

### Statistical analysis

The normality of data was tested by the Shapiro-Wilk test. For the comparison of two groups, we used the t-test to detect the significant difference between normally distributed data, and the Wilcoxon test for skewed distributed data. For the comparison of three or more groups, one-way ANOVA and Kruskal-Wallis tests were conducted to detect the significant difference between normal distributed and skewed distributed data, respectively. A chi-squared test was used to compare the frequency differences between the two groups. Correlation coefficients were calculated by the Spearman and distance correlation analyses.

For the survival analysis, we focused on the overall survival (OS) and recurrence-free survival (RFS), and we obtained the best cut-off value through the “*survcutpoint*” function of the “*survminer*” R package. Kaplan-Meier method was used to depict the survival curves, and the log-rank test was utilized to identify significant survival differences between groups. Univariate Cox proportional hazards regression model was applied to calculate the hazard ratio (HR) and 95% confidence interval (95% CI) for each DNA methylation regulator and DNA methylation related gene. And the multivariate Cox model was performed to determine the independent prognostic factors when adjusted by clinical characteristics. The results of Cox regression analyses were visualized by the “*forestplot*” R package. The prediction performance of DMS to evaluate the OS probability at distinct times was assessed by the receiver operating characteristic (ROC) curves and quantified by the area under the curve (AUC), which were conducted *via* the “*timeROC*” R package.

All statistical analyses were accomplished in R 3.6.1 software, and all reported *p*-values were two-sided, with *p*-value < 0.05 as statistically significant.

## Results

### Multi-omics landscape analysis of DNA methylation regulators in colon cancer

**FIGURE 1:**
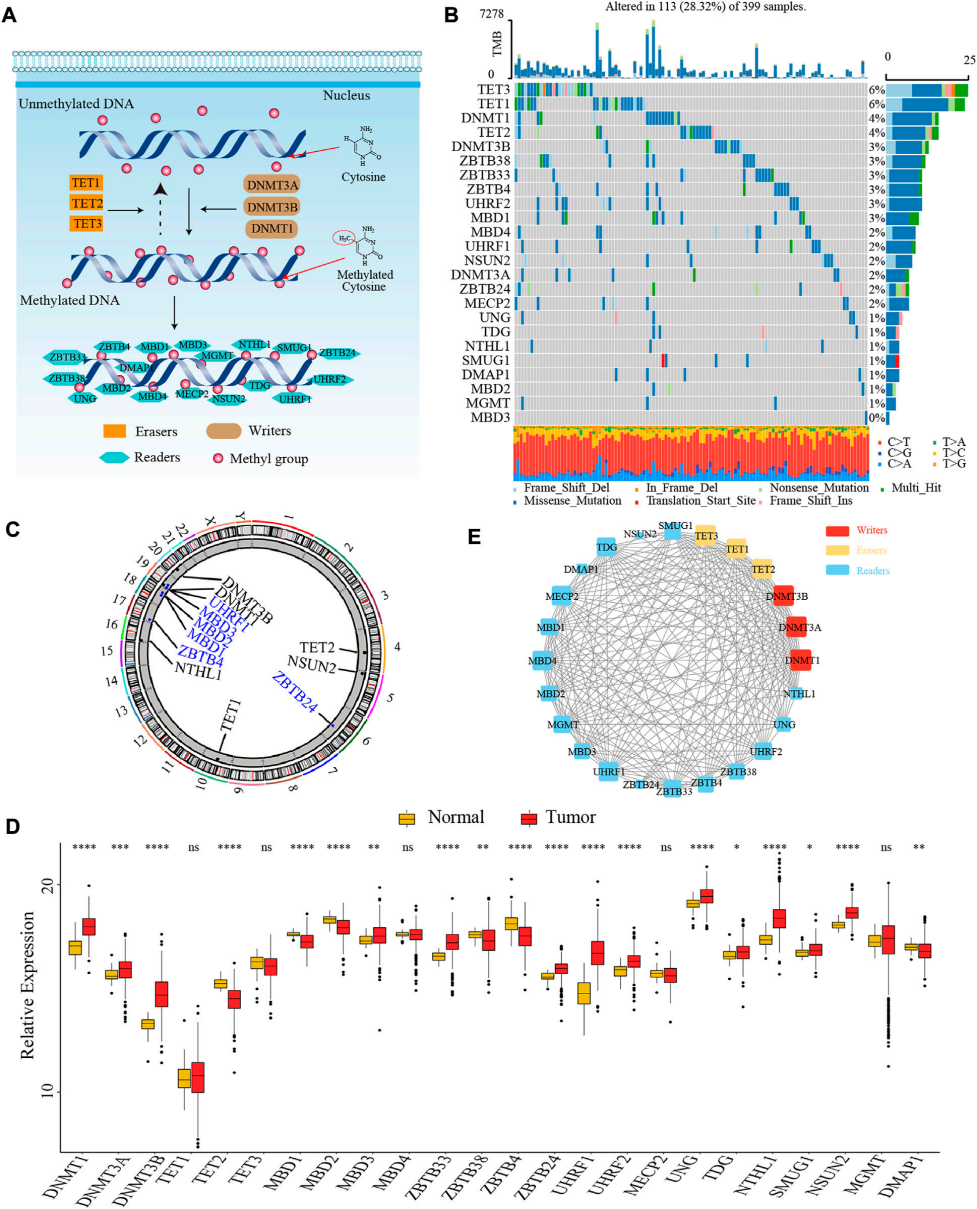
The landscape of genetic and transcriptomic variations of 24 DNA methylation regulators in colon cancer. **(A)** The dynamic reversible DNA methylation modification process of 24 regulators. **(B)** The mutation frequency of 24 DNA methylation regulators in the TCGA-COAD cohort. **(C)** The location of CNV alteration of DNA methylation regulators on 23 chromosomes from TCGA-COAD cohort. **(D)** The expression of 24 DNA methylation regulators between colon cancer tissue and normal tissue (Wilcoxon test; ns: no significance; *p < 0.05; **p < 0.01; ***p < 0.001; ****p < 0.0001). **(E)** The PPI network among 24 DNA methylation regulators.

In this study, we identified 24 DNA methylation regulators, including 3 writers, 3 erasers, and 18 readers. Figure 1A summarized the dynamic reversible DNA methylation modification process of these regulators. Firstly, we analyzed the incidence of somatic mutation and CNV of 24 regulators in colon cancer from the genomic perspective. We visualized the mutation landscape of 24 regulators through the “*waterfall*” function of the “*maftools*” R package. Among 399 available samples in the TCGA-COAD cohort, a total of 113 (28.3%) mutations occurred in 24 regulators (Figure 1B). Therein, 2 erasers, TET3 and TET1, had the highest mutation frequency (6%) with missense mutation as a major mutation type, while MBD3 did not present any mutations. Besides, we found significant co-occurrence mutation relationships between several regulators, such as TET3 and ZBTB38, DNMT1 and MBD1, along with UHRF2 and MECP2 (Supplementary Figure S1B). In addition, the CNV analysis revealed an amplification of copy number in 6 regulators and deletion in other 6 regulators. The location of CNV alterations of 12 regulators on the chromosomes was shown in Figure 1C, which was accomplished by the “*RCircos*” R package.

Secondly, to ascertain whether the above genomic variations affected the transcription of 24 regulators, we further compared the mRNA expression level of these regulators between colon cancer and normal samples from the transcriptomic perspective. We observed most of the regulators were significantly differentially expressed between tumor and normal tissues. Interestingly, compared to normal tissues, we found that many regulators with amplified CNV had a markedly higher expression level in colon cancer tissues (e.g., DNMT1 and DNMT3B), and vice versa (e.g., MBD2 and ZBTB4), suggesting that the alteration of CNV might be the prominent factor resulting in the abnormal expression of DNA methylation regulators (Figures 1C,D). To further confirm the expression level of these regulators in colorectal cancer cell line and normal colonic epithelial cell line, we selected three most significantly differentially expressed regulators between tumor and normal tissues, DNMT3B (writer), TET2 (eraser), and UHRF1 (reader), to compare their mRNA expression in HCT116 and NCM460 cells. The results of quantitative reverse transcription-polymerase chain reaction (qRT-PCR) showed that the mRNA level of DNMT3B and UHRF1 were significantly higher in HCT116 cells while the mRNA level of TET2 was significantly higher in NCM460 cells (Supplementary Figures S1C–E).

Thirdly, we explored the interaction relationship between 24 regulators from the proteomics perspective. The PPI network depicted the extensive protein interactions among these regulators (Figure 1E). Comprehensively, the above analyses presented a highly genetic heterogeneity and expressional anomalism of DNA methylation regulators in colon cancer from a multi-omics landscape perspective, hinting at a critical role of these regulators in tumorigenesis of colon cancer.

### Prognosis and immune characteristics of 24 DNA methylation regulators in colon cancer

**FIGURE 2:**
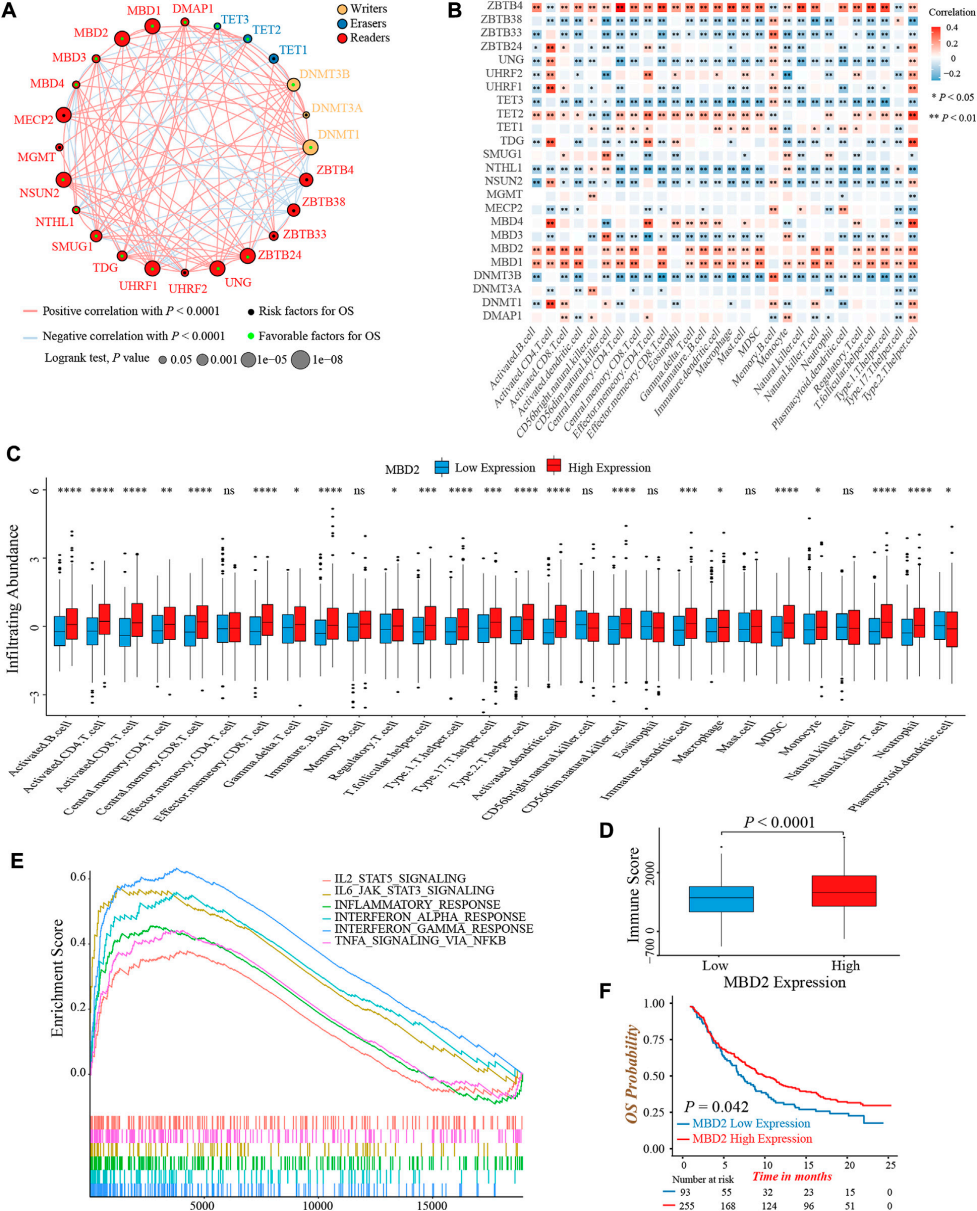
Prognosis and immune characteristics of 24 DNA methylation regulators in colon cancer. **(A)** The prognosis and correlation among 24 DNA methylation regulators in colon cancer (Log-rank test and Spearman correlation analysis). **(B)** The correlation between 28 TME infiltrating cell types and 24 DNA methylation regulators (Spearman correlation analysis). **(C)** The infiltrating abundance of 28 TME cell types between the MBD2 high and low expression groups (Wilcoxon test; ns: no significance; *p < 0.05; **p < 0.01; ***p < 0.001; ****p < 0.0001). **(D)** Comparison of immune score between the MBD2 high and low expression groups. (Wilcoxon test; *p* < 0.0001). **(E)** GSEA analysis indicated six activated immune/inflammation-related pathways that were enriched in the MBD2 high expression group (All *p* < 0.05). **(F)** Overall survival analysis of high and low MBD2 expression groups in the IMvigor210 cohort (Log-rank test, *p* = 0.042).

To clarify the role of 24 DNA methylation regulators in colon cancer clinical prognosis and TME cell infiltration characterization, we gathered three GEO datasets (GSE39582, GSE38832, and GSE39084) without prognostic differences as meta-cohort for further analyses (Supplementary Figure S2A). The Cox regression analysis identified high expression of nearly half of the regulators were associated with a favorable prognosis (Supplementary Figure S2B). Moreover, the interaction network of regulators visualized the correlation of regulators’ expression and their prognostic significance (Figure 2A; Supplementary Table S5). We found that the expression of most regulators was positively related to each other, not only in regulators with the same functional category, but also among writers, erasers, and readers.

TME infiltrating immune cells had been widely reported to display an epigenomic reprogramming, especially in aberrant DNA methylation (Loo Yau et al., 2019). Therefore, we investigated the correlation between 24 regulators and TME infiltrating cells. The expression of MBD1, MBD2, and ZBTB4 was positively correlated with infiltrating abundance of majority immune cells, which could explain their favorable prognostic value to some extent (Figure 2B). A previous study demonstrated that colon cancer patients with a MSI status presented an active intra-tumoral immune environment, and hence, had a robust response to immunotherapy and superior prognosis (Andre et al., 2020). Similarly, we found the expression of MBD1 and MBD2 was upregulated in MSI colon cancer patients, implying both regulators might involve in immune activation (Supplementary Figure S2C). In consideration of the MBD2 expression were highly correlated to immune cells quantity, especially for activated CD4+ and CD8+ T-cells, as well as its preferable prognostic value, we next thoroughly analyzed the role of MBD2 in tumor immune environment formation. First, the MBD2 high expression group exhibited a higher infiltrating abundance of most immune cells and TME immune score (Figures 2C,D). Second, GSEA analysis revealed several activated immune and inflammation-related pathways, such as the TNFα, interferon- and interleukin-mediated signaling pathways were significantly enriched in the MBD2 high expression group (Figure 2E). Third, owing to the vital role of MBD2 in tumor immunity, we additionally investigated whether its expression could predict the efficacy and prognosis of immunotherapy. In the IMvigor210 cohort, we observed a marked survival benefit in patients with MBD2 high expression, although the therapeutic response was similar between two groups (Figure 2F; Supplementary Figure S2D).

Collectively, above results exhibited crosstalk among 24 DNA methylation regulators and their significant impact on tumor immunity. The MBD2 expression was positively correlated to TME immune cells infiltration and might be a potential prognostic biomarker in immunotherapy.

### DNA methylation modification patterns mediated by 24 regulators in colon cancer

**FIGURE 3:**
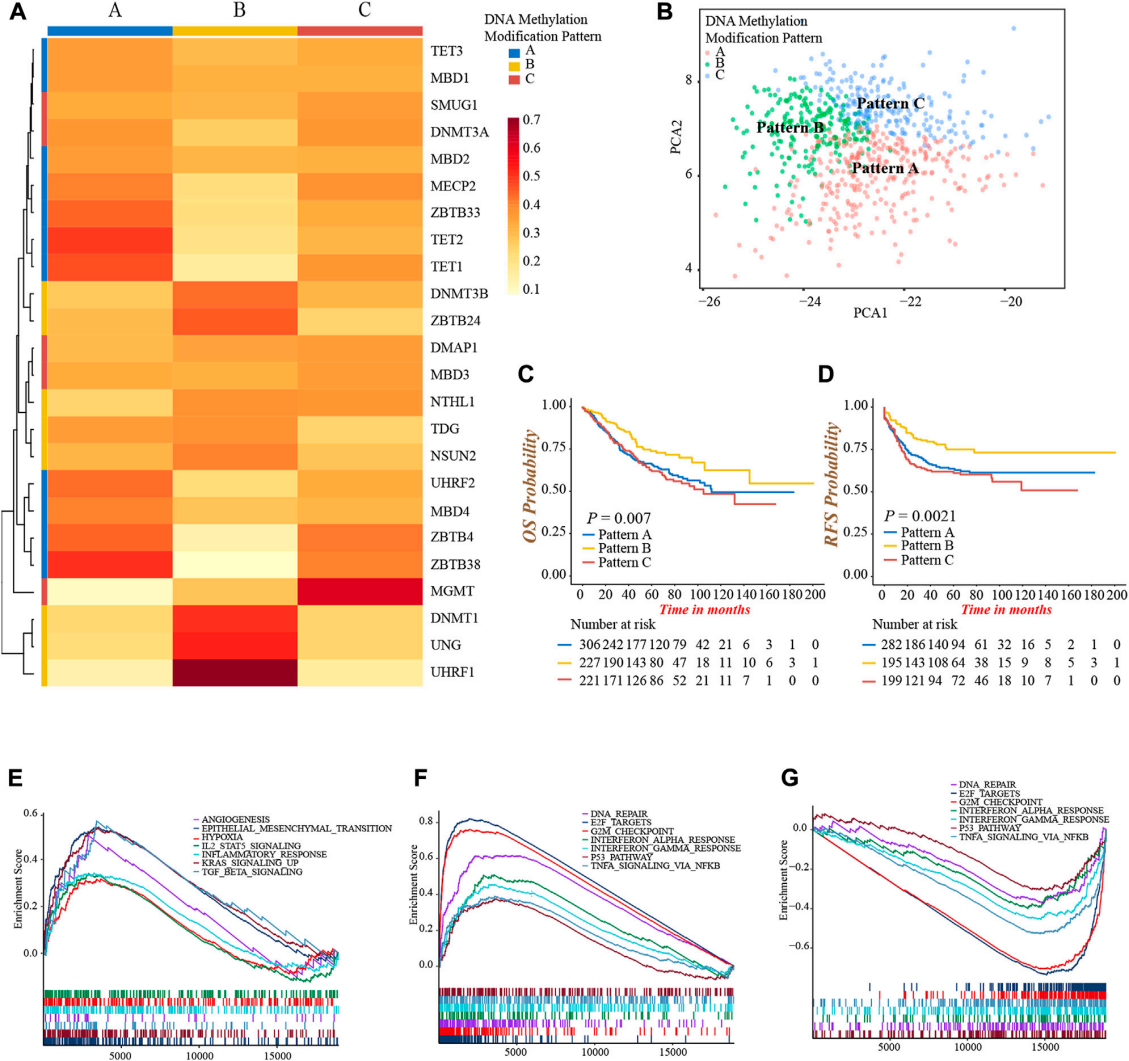
Identification of DNA methylation modification patterns and prognostic and pathway analyses. **(A)** Unsupervised clustering of 24 DNA methylation regulators in meta-cohort. **(B)** PCA graph for the transcriptomic profiles of three DNA methylation modification patterns. **(C)** Overall survival analysis for the three DNA methylation modification patterns (Log-rank test, *p* = 0.007). **(D)** Recurrence-free survival analysis for the three DNA methylation modification patterns (Log-rank test, *p* = 0.0021). **(E–G)** GSEA enrichment analyses presented the activation status of biological pathways among distinct DNA methylation modification patterns. **(E)** Pattern-A; **(F)** Pattern-B; **(G)** Pattern-C.

As the markedly different clinical outcomes and TME infiltrating cells characterizations among 24 DNA methylation regulators in colon cancer, we speculated that these regulators may contribute to forming different DNA methylation modification patterns in individual colon cancer. Based on the expression of 24 regulators, we applied the NMF unsupervised clustering to obtain three distinct clusters, including DNA methylation modification pattern-A (*n* = 306), pattern-B (*n* = 227), and pattern-C (*n* = 221), in meta-cohort (Supplementary Figures S3A,B). Accordingly, we found different regulators were differentially expressed in different patterns. Pattern-A was characterized by high expression of TET1, TET2, ZBTB4, ZBTB33, ZBTB38, MBD4, UHRF2, and MECP2; pattern-B was characterized by high expression of DNMT1, DNMT3B, ZBTB24, UHRF1, and UNG; and pattern-C was characterized by high expression of DMAP1, SMUG1 and MGMT (Figure 3A; Supplementary Figure S3C). In addition, the graph of principal component analysis (PCA) showed that three patterns were distinctly segregated, indicating a complete distinguishment of three patterns (Figure 3B). Moreover, the survival analysis revealed a notably favorable OS and RFS in pattern-B (Figures 3C,D).

Then, we performed a GSEA analysis to explore the different hallmark signaling pathways among three patterns (Supplementary Table S6). We found pattern-A presented the activated enrichment pathways associated with stromal and carcinogenic activation, such as the KRAS, TGFβ, EMT, and angiogenesis pathways, as well as the highest stromal score (Figure 3E; Supplementary Figure S3D). Immune regulation and tumor suppressor-related pathways were significantly enriched in pattern-B, including interferon-α, and -γ, TNFα, P53 signaling, G2M checkpoint, and DNA damage repair related pathways (Figure 3F). In contrast, these pathways were all inhibited in pattern-C (Figure 3G). We subsequently compared the abundance of TME infiltrating cells among three patterns. Not surprisingly, high infiltration of activated CD4+ and CD8+ T-cells were observed in pattern-B (Supplementary Figure S3E; Supplementary Table S7), which was consistent with the results of enrichment into activated immune-related pathways, and may explain the preferable prognosis (Figures 3C,D). Meanwhile, several innate immune cells were remarkably enriched in pattern-A, including eosinophil, mast cell, and plasmacytoid dendritic cell, while the content of the majority of immune cells was relatively lower in pattern-C (Supplementary Figure S3E).

From the above analyses, we were surprised to find three DNA methylation modification patterns exhibited significantly distinct biological behaviors and TME infiltrating cell characterizations, in accordance with the conception of three tumor immune phenotypes (Hegde and Chen, 2020). Pattern-A was classified as immune-excluded phenotype, characterized by innate immune cell infiltration and stromal activation; pattern-B was classified as immune-inflamed phenotype, characterized by a high population of adaptive immune cell infiltration and immune activation; pattern-C was classified as immune-desert phenotype, characterized by few infiltrations of immune cells and immune suppression.

### Generation of DNA methylation gene signature

**FIGURE 4:**
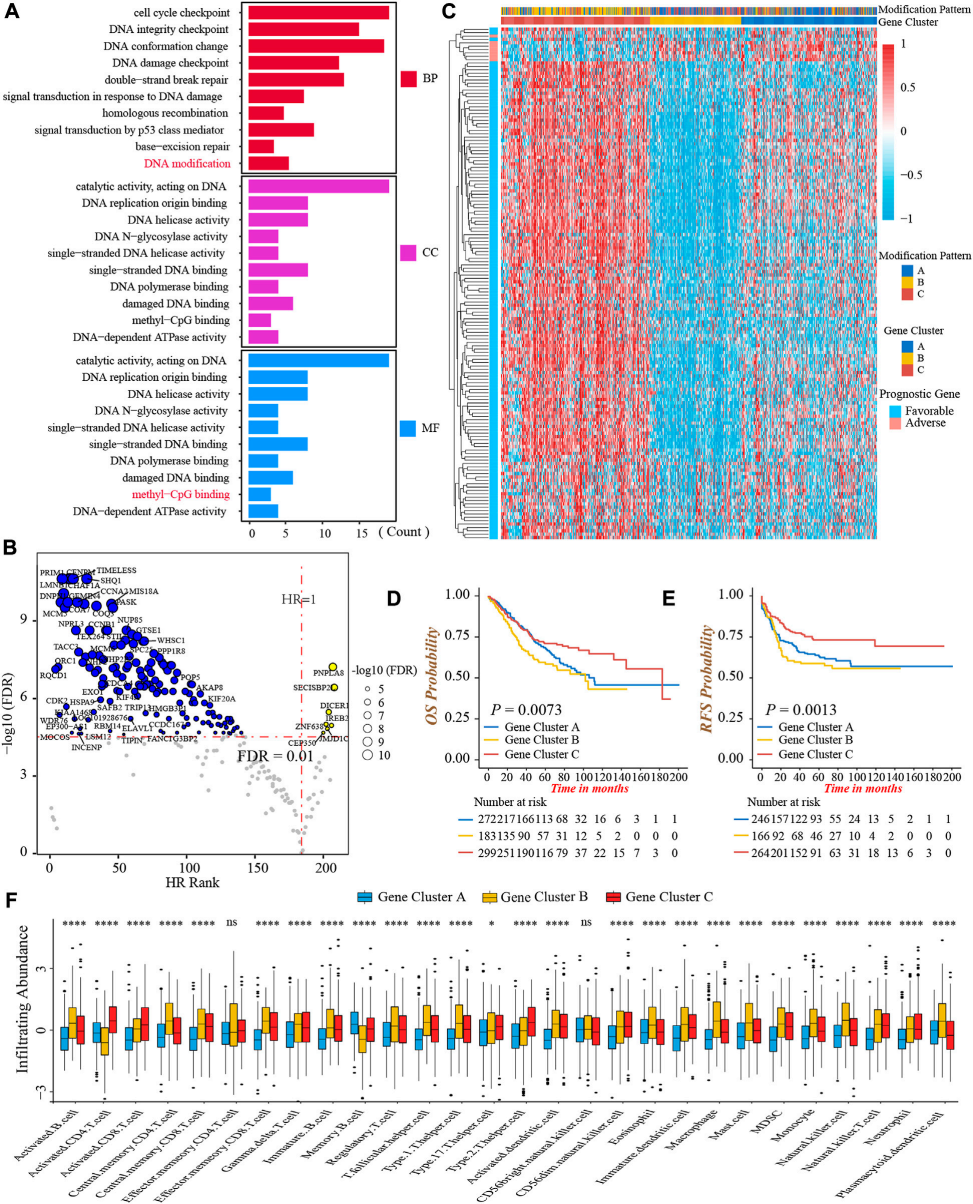
Generation of DNA methylation gene signature. **(A)** Functional annotation of 249 DEGs among three DNA methylation modification patterns using GO enrichment analysis. BP, biological process; CC, cellular component; MF, molecular function. **(B)** Volcano Plot presented the DEGs significantly associated with prognosis. **(C)** The heatmap of 152 prognostic DEGs’ expressions among three DNA methylation gene clusters-A, -B, and -C. **(D)** Overall survival analysis for the three DNA methylation gene clusters (Log-rank test, *p* = 0.0073). **(E)** Recurrence-free survival analysis for the three DNA methylation gene clusters (Log-rank test, *p* = 0.0013). **(F)** The infiltrating abundance of 28 TME cell types among three DNA methylation gene clusters (Kruskal-Wallis test; ns, no significance; *p < 0.05; **p < 0.01; ***p < 0.001; ****p < 0.0001).

To further investigate the heterogeneity of three DNA methylation modification patterns, we identified 249 DEGs among three patterns (Supplementary Figures S4A,B). The GO functional annotation showed that these DEGs were significantly enriched in pathways related to DNA modification and damage repair events (Figure 4A; Supplementary Table S8), indicating the differences in clinical outcomes and TME characterizations among three patterns might result from these DEGs. Subsequently, we obtained 152 genes associated with prognosis through univariate Cox regression analysis (Figure 4B; Supplementary Table S9). Furthermore, based on the expression of 152 genes, we conducted unsupervised clustering to classify patients into different genomic subtypes. Surprisingly, consistent with three modification patterns, the NMF algorithm still clustered three distinct DNA methylation genomic phenotypes, named DNA methylation gene cluster-A (*n* = 272), cluster-B (*n* = 183), and cluster-C (*n* = 299), respectively (Figure 4C; Supplementary Figures S4C,D). The plot of PCA also presented a distinct separation of three gene clusters (Supplementary Figure S4E). As well, the prominent differences in the expression of 24 DNA methylation regulators were observed among three gene clusters, in line with the results of three modification patterns (Supplementary Figure S4F). In accordance with the feature of modification pattern-B, gene cluster-C had a superior prognosis and the highest CD8+ T-cells infiltrating abundance than other two clusters, demonstrating gene cluster-C was immune-inflamed phenotype (Figures 4D–F). Above all, these analyses verified the perspective that there were indeed three disparate immune phenotypes in colon cancer, representing different clinical and TME immune characterizations.

### Characterization of DNA methylation score in different clinical and molecular traits

The above results demonstrated DNA methylation modification played a non-negligible role in TME formation and prognosis of colon cancer patients. Nevertheless, the remarkable heterogeneity between individual tumors limited the accurate application of DNA methylation modification patterns. Therefore, based on 152 prognostic DEGs, we constructed a DNA methylation gene signature, termed as DNA methylation score (DMS) to quantify the DNA methylation modification pattern of each patient.

**FIGURE 5:**
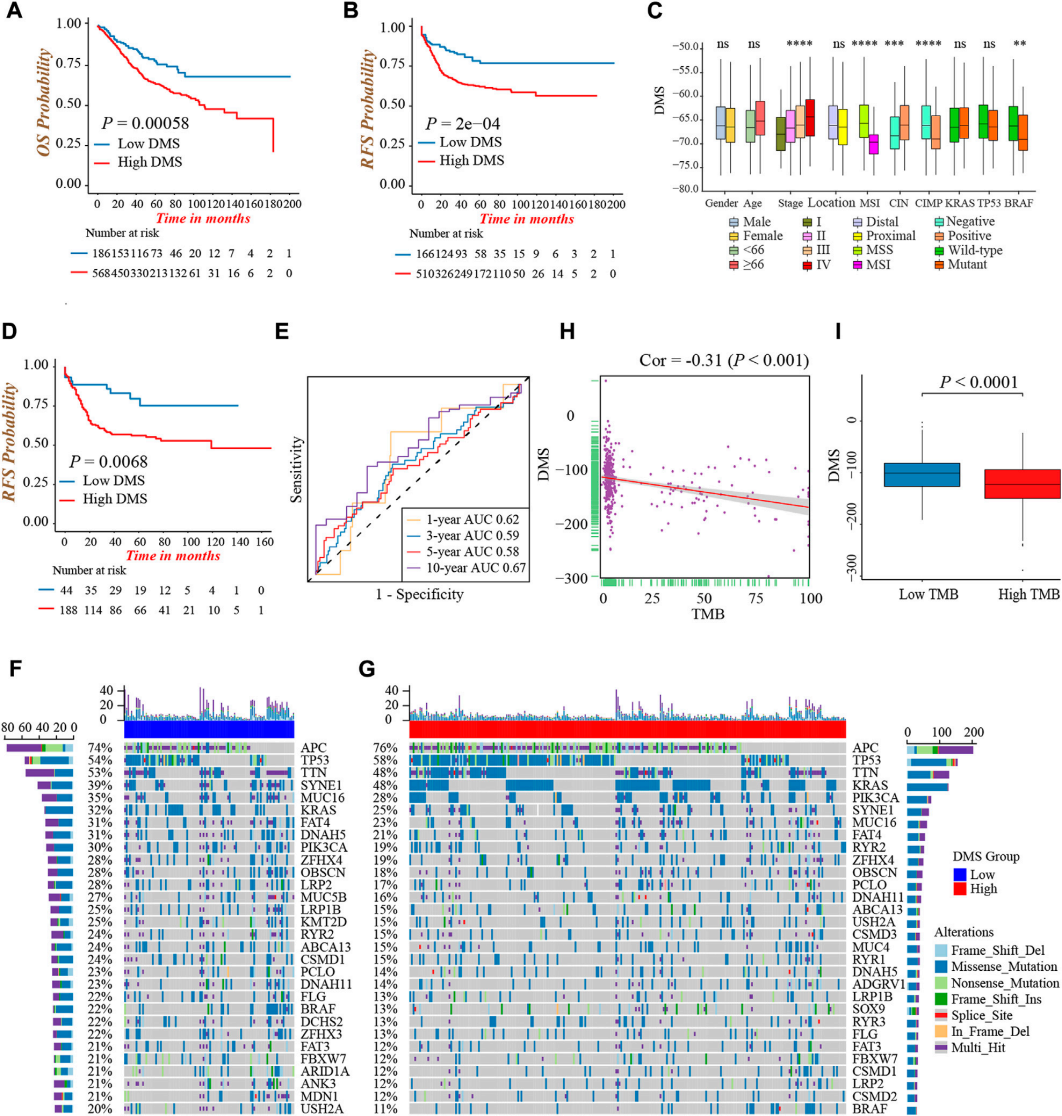
Characterization of DNA methylation score in different clinical and molecular traits. **(A)** Overall survival analysis for low and high DMS groups in the meta-cohort (Log-rank test, *p* = 0.00058). **(B)** Recurrence-free survival analysis for low and high DMS groups in the meta-cohort (Log-rank test, *p* = 0.0002). **(C)** Difference in DMS among distinct clinical subgroups in the GSE39582 cohort. MSI, microsatellite instability; CIMP, CpG island methylator phenotype; CIN, chromosome instability. (Wilcoxon test; ns: no significance; *p < 0.05; **p < 0.01; ***p < 0.001; ****p < 0.0001). **(D)** Recurrence-free survival analysis for low and high DMS groups among patients received chemotherapy in the GSE39582 cohort (Log-rank test, *p* = 0.0068). **(E)** ROC curves to reflect the ability of DMS to predict the 1-, 3-, 5-, and 10-year survival probability for patients received chemotherapy in the GSE39582 cohort. **(F,G)** The waterfall plot presented the distribution differences of tumor somatic mutation between DMS-low **(F)** and -high groups **(G)**. **(H)** The scatter plot depicted the negative correlation between DMS and TMB in the TCGA-COAD cohort (Spearman correlation analysis; *p* < 0.001). **(I)** Comparison of DMS between the TMB high and low groups (Wilcoxon test; *p* < 0.0001).

We first evaluated the prognostic value of DMS in colon cancer, and determined −73.8 as the cut-off value to divide patients into low and high DMS groups. Prognostic analysis showed patients with low DMS had a prominently prolonged survival and recurrence-free time (Figures 5A,B). Besides, the multivariate Cox regression analysis demonstrated DMS was an independent risk prognostic biomarker for colon cancer (HR was 1.70 for OS and 1.66 for RFS; Supplementary Figure S5A). Additionally, to detect the stability of DMS, we enrolled other independent colon cancer cohorts to validate the prognostic value of DMS. Likewise, the DMS-low group had a preferable survival in the TCGA-COAD cohort, GSE72970, GSE103479, and GSE87211 cohorts (all *p* < 0.05, Supplementary Figures S5B–G). Furthermore, we continued to expand the application of DMS in other digestive system tumors, and we found rectum adenocarcinoma and stomach adenocarcinoma patients with low DMS presented a remarkably longer survival (both *p* < 0.05, Supplementary Figures S5H,I). The data above proved that the DMS we constructed could predict the survival of colon cancer effectively and stably.

Next, we explored the characteristics of DMS in different clinical and molecular subtype traits and fixed our attention on the GSE39582 cohort, which had comprehensive clinical information. We found DMS rose gradually by increasing the tumor TNM stage (*p* < 0.001, Figure 5C). Besides, patients with MSI, CIMP positive, CIN negative, and mutant BRAF showed a lower DMS, implying these subtypes may have a better prognosis (all *p* < 0.05, Figure 5C). Chemotherapy was a standard treatment strategy for colon cancer, and hence, we specifically examined the ability of DMS to predict the efficacy of chemotherapy. We revealed significantly longer recurrence-free time in the DMS-low group of colon cancer patients undergoing chemotherapy (Figure 5D), and the ROC curves to reflect the ability of DMS to predict the 1-, 3-, 5-, and 10-year survival probability were shown in Figure 5E.

Then, we investigated the distribution differences of somatic mutation between DMS-low and -high groups. As shown in Figures 5F,G, the DMS-low group exhibited a higher mutation frequency, and APC, TP53, and TTN were three common genes with the highest mutation frequency in both groups. As well, we quantified tumor mutation burden (TMB) value, and discovered a markedly negative correlation between DMS and TMB (Figure 5H). Same as the relation between MSI status and DMS, the TMB-high group presented a lower DMS, hinting low DMS may reflect genomic instability, massive tumor neoantigen generation, and induce immune activation (Figure 5I). Generally, these accumulated results manifested DMS was an effective predictive tool, and distinguished different molecular subtypes of colon cancer.

### The role of DNA methylation score in the tumor microenvironment and biological processes

**FIGURE 6:**
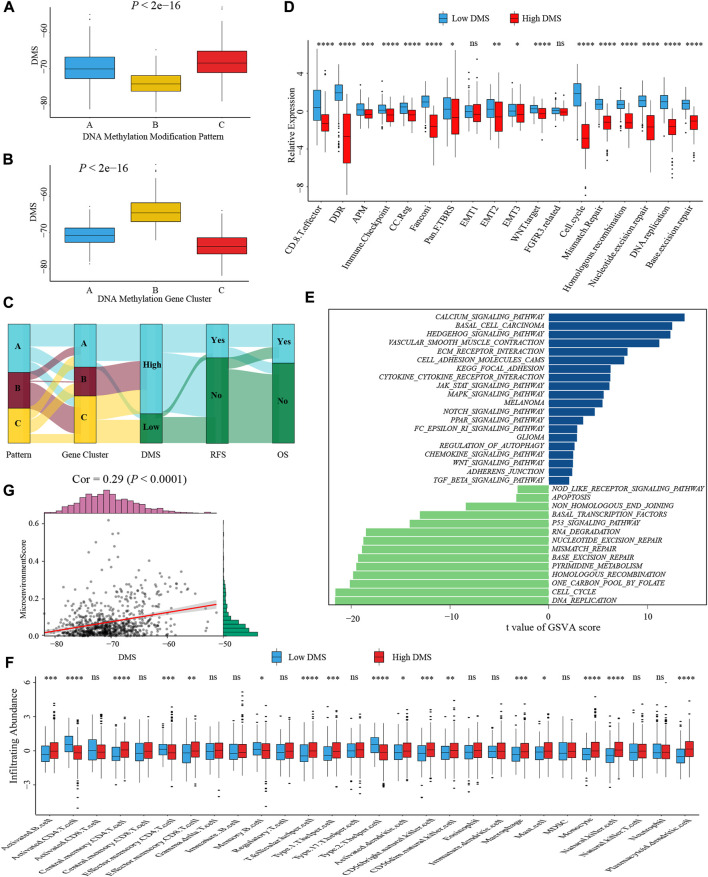
Characterization of DMS in TME and biological processes. **(A)** Differences in DMS among three DNA methylation modification patterns in the meta-cohort (Kruskal-Wallis test; *p* < 0.001). **(B)** Differences in DMS among three DNA methylation gene clusters in the meta-cohort (Kruskal-Wallis test; *p* < 0.001). **(C)** Sankey diagram visualized the distribution of individual colon cancer patients in different DNA methylation modification patterns, gene clusters, DMS, and clinical outcomes. **(D)** The differences in biological processes signatures between DMS-high and -low groups (Wilcoxon test; ns: no significance; *p < 0.05; **p < 0.01; ***p < 0.001; ****p < 0.0001). **(E)** GSVA analysis of relatively activated KEGG pathways between DMS-high and -low groups. Blue bars represented activated pathways in the DMS-high group, and green bars represented activated pathways in the DMS-low group. **(F)** The ssGSEA method identified infiltrating abundance of 28 TME cell types between DMS-high and -low groups (Wilcoxon test; ns: no significance; *p < 0.05; **p < 0.01; ***p < 0.001; ****p < 0.0001). **(G)** The scatter plot depicted the positive correlation between DMS and microenvironment score (Spearman correlation analysis; *p* < 0.0001).

To better illustrate the underlying relevance between DMS and different molecular traits of colon cancer, we first compared DMS among different DNA methylation modification patterns and gene clusters. Pattern-B and gene cluster-C, representing the immune-inflamed phenotype, both had the lowest median DMS (Figures 6A,B). A similar distribution trend of DMS among three patterns and gene clusters was shown in Supplementary Figures S6A,B when we split the meta-cohort into three separate GSE cohorts. These results verified low DMS was related to better survival again, and the Sankey diagram visualized the distribution of individual colon cancer patients in different DNA methylation modification patterns, gene clusters, DMS, and clinical outcomes (Figure 6C).

Subsequently, we analyzed the correlation between DMS and several known biological processes signatures constructed by Mariathasan et al. (2018). The results revealed DMS was mightily negatively correlated with DNA damage repair-related signatures (Supplementary Figure S6C). Further comparative analysis demonstrated a higher activity of immune and DNA damage repair pathways in the DMS-low group, including CD8+ T effector, immune checkpoint, and mismatch repair (Figure 6D). Additional GSVA analysis revealed activated DNA damage repair and tumor suppresser-related pathways were significantly enriched in the DMS-low group, while stromal and carcinogenic activation pathways were markedly enriched in the DMS-high group (Figure 6E). Above analyses confirmed DNA methylation patterns participated in these biological processes and the DMS could reflect the activity of immune and stromal-related pathways.

We then examined the relationship between TME infiltrating cells and DMS using different immunocytes associated algorithms, including ssGSEA, CIBERSORT, and TIMER database, and we found the majority of immune cells, especially CD8+ T cells, presented a high infiltrating abundance in the DMS-low group (Figure 6F; Supplementary Figures S6G,H). Besides, the tumor microenvironment score, estimate score, and stromal score showed a positive correlation with DMS (Figure 6G; Supplementary Figures S6D,E). Furthermore, we compared the expression level of 24 DNA methylation regulators between DMS-low and -high groups and revealed that some regulators positively associated with immune cells were also highly expressed in the DMS-low group, such as TET2, MBD1, and MBD2 (Supplementary Figure S6F). At last, we investigated several molecules, including chemokines and cytokines associated with immune activation or suppression, which were referenced from published literature (Zeng et al., 2019). Of these molecules, CD8A, CXCL10, CXCL9, GZMA, GZMB, IFNG, PRF1, TBX2, and TNF were considered to be correlated to the transcripts of immune activation; and CD80, CD86, CTLA-4, HAVCR2, IDO1, LAG3, PD-1, PD-L1, PD-L2, TIGIT, and TNFRSF9 were related to the transcripts of immune checkpoints. We still found the expression of mRNAs relevant to immune activation was significantly upregulated in the DMS-low group (Supplementary Figure S6I). While two immune checkpoints, PD-1 and PD-L2, were highly expressed in the DMS-high group (Supplementary Figure S6J). Collectively, these results demonstrated DNA methylation involved in TME immunity response. Then combining the above conclusions that low DMS was related to higher TMB, we speculated that DNA methylation modification might influence the genetic mutation and generate a great number of tumoral neoantigens, inducing immune activation. These results provided a novel perspective to explore the mechanism of DNA methylation in tumor somatic mutation, and indirectly hinting DMS may play an important role in tumor immunotherapy.

### The predictive value of DNA methylation score in anti-PD-1/PD-L1 immunotherapy

**FIGURE 7:**
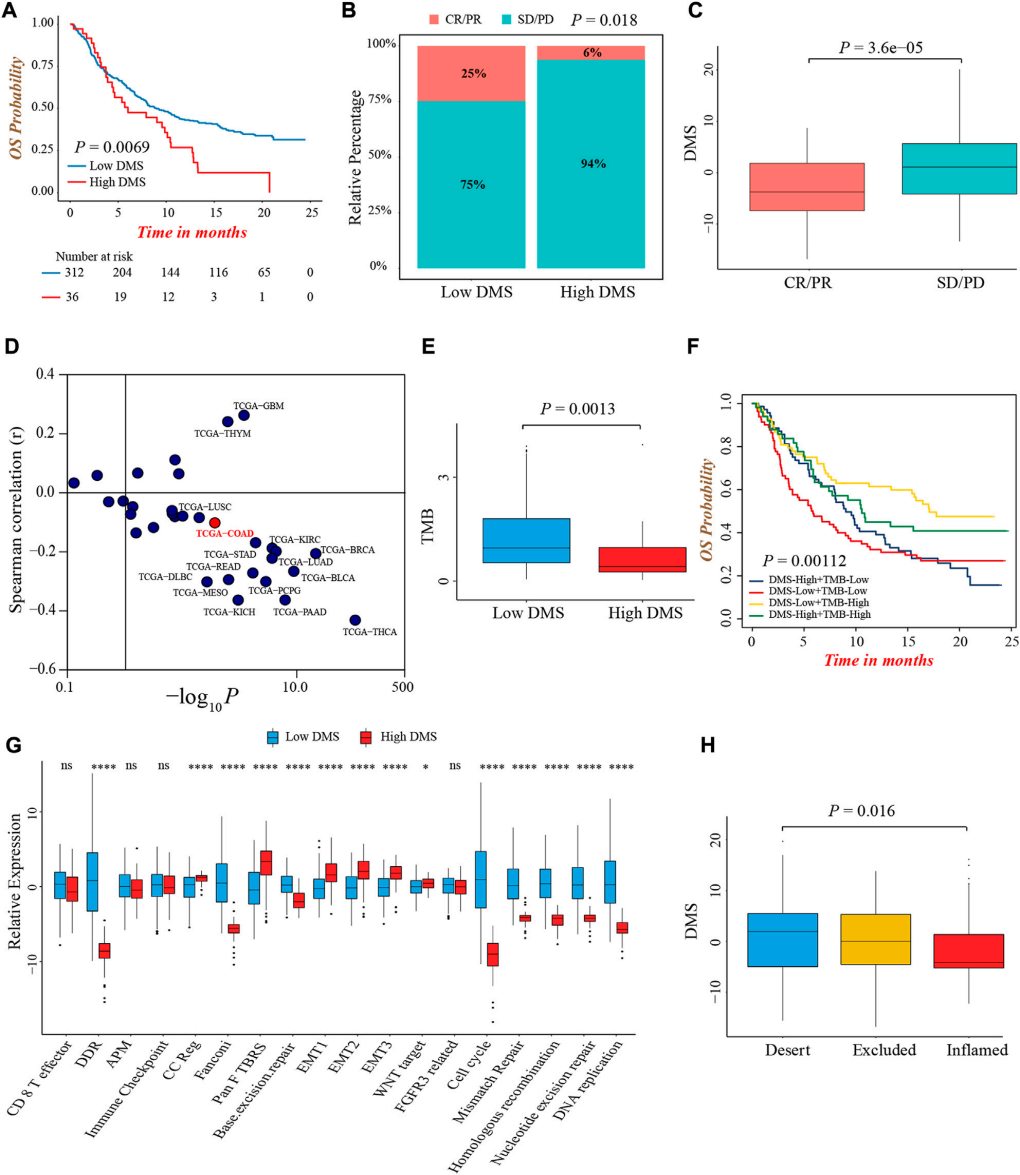
The predictive value of DMS in immunotherapy. **(A)** Overall survival analysis for low and high DMS groups in the IMvigor210 cohort (Log-rank test, *p* = 0.0069). **(B)** The proportion of patients with response to immunotherapy between DMS-high and -low groups in the IMvigor210 cohort (Chi-square test, *p* = 0.018). SD, stable disease; PD, progressive disease; CR, complete response; PR, partial response. **(C)** Comparison of DMS between immunotherapy response and non-response groups in the IMvigor210 cohort (Wilcoxon test; *p* < 0.0001). **(D)** Correlations between DMS and PD-L1 expression in pan-cancer cohorts through Spearman analysis (spearman correlation analysis). **(E)** Comparison of TMB between DMS-high and -low groups in the IMvigor210 cohort (Wilcoxon test; *p* = 0.0013). **(F)** Survival analyses for subgroup patients stratified by both DMS and TMB in the IMvigor210 cohort (Log-rank test; *p* = 0.00112). **(G)** The differences in biological processes signatures between DMS-high and -low groups in the IMvigor210 cohort (Wilcoxon test; ns: no significance; *p < 0.05; **p < 0.01; ***p < 0.001; ****p < 0.0001). **(H)** Differences in DMS among distinct tumor immune phenotypes in the IMvigor210 cohort. (Kruskal-Wallis test; *p* = 0.016).

Immunotherapy represented by anti-PD-1/PD-L1 or CTLA-4 antibody had broadened the field of cancer treatment and brought huge clinical benefits in recent years. Here, we explored whether DMS could predict the therapeutic response and prognosis of patients treated with immunotherapy. In the IMvigor210 cohort, the DMS-low group presented a remarkably prolonged survival, and the multivariate Cox regression analysis also determined that higher DMS was an independent risk factor for prognosis (Figure 7A; Supplementary Figure S7A). The ROC curves for DMS in predicting 12-, 18-, 24-months survival probability were presented in Supplementary Figure S7B. The objective response rate (percentage of complete response and partial response) of patients with low DMS was significantly higher (Figure 7B). As well, patients in the response group showed a lower DMS compared to the non-response group, indicating a clinical benefit and treatment advantage of immunotherapy in patients with low DMS (Figure 7C; Supplementary Figure S7C).

Although we did not find a significant difference in PD-L1 expression level, a potential biomarker for immunotherapy, between DMS-low and -high groups in IMvigor210 cohort (Supplementary Figure S7D), the pan-cancer analysis demonstrated DMS was negatively related to PD-L1 and PD-L2 expression in the majority of cancer types including colon cancer, indirectly proving the reliability of DMS to predict the efficacy of immunotherapy (Figure 7D; Supplementary Figure S7E). In this immunotherapeutic cohort, the TMB value was also higher in the DMS-low group, which confirmed our above conclusions again (Figure 7E). In addition, we evaluated the prognostic value of TMB and DMS combination in immunotherapy, and observed a marked prognosis benefit in patients with low DMS and high TMB (Figure 7F).

More importantly, the biological processes signatures analyses showed that DNA damage repair-related pathways were significantly activated in the DMS-low group, while EMT and Pan-F-TBRS pathways were highly activated in the DMS-high group, indicating DMS was closely related to the DNA damage repair and stromal signatures in the setting of patients receiving immunotherapy (Figure 7G). Lastly, we investigated the difference of DMS among different immune phenotypes identified by the IMvigor210 study, and found the immune-excluded and -desert phenotypes had higher DMS, implying the response to immunotherapy of these subtypes was limited (Figure 7H). In summary, our work demonstrated a significant correlation between DNA methylation modification patterns and tumor immune phenotypes, and DMS could help to predict the response of immunotherapy.

## Discussion

In this study, based on 24 DNA methylation regulators, we first identified three DNA methylation modification patterns with distinct TME infiltrating characteristics and biological behaviors in colon cancer. Moreover, we obtained prognostic DEGs among three modification patterns and established the DNA methylation gene signature, termed DMS, to quantify the DNA methylation modification profile of individual colon cancer, and more importantly, predict the efficacy and clinical outcome of immunotherapy.

Increasing evidence revealed tumors commonly hijacked various epigenetic mechanisms to escape the supervision of the immune system. Particularly, certain regulator mediated DNA methylation and demethylation played an indispensable role in adaptive immune response, including generation of tumoral neoantigen, dysregulation of antigen-presenting machinery, and suppression of anti-tumor cytokine production (Cao and Yan, 2020). However, the analysis of the whole landscape of DNA methylation modification in colon cancer was limited, and its impact on TME immune response remained unclear. Here, we integrated the transcriptomic information of 24 DNA methylation regulators and revealed three distinct DNA methylation modification patterns, and their features were consistent with three classical tumor immunophenotypes: immune-inflamed, -excluded, and -desert (Hegde and Chen, 2020). Modification pattern-B was immune-inflamed phenotype and presented an activated immune status, characterized by activation of immune, tumor suppressor and DNA damage repair related pathways, as well as a high infiltrating abundance of activated CD8+ T cell. Accordingly, patients in pattern-B had a better prognosis than the other two patterns. In detail, abundant immune cells were positioned in proximity to the tumor cells, accompanied by many proinflammatory and effector chemokines and cytokines, suggesting the presence of pre-existing anti-tumor immunity in the tumor parenchyma (Harlin et al., 2009; Gajewski et al., 2013; Herbst et al., 2014). Mechanically, an inflammatory TME was the basis of immune-inflamed phenotype, also known as a hot tumor, containing pro-inflammatory cytokines which provided a more favorable condition for T cell activation and expansion, including type I and type II interferons, TNF-α, IL-2, and IL-12. By contrast, pattern-C was immune-desert phenotype and exhibited an immune suppression status, characterized by inhibition of multiple immune and inflammatory-related pathways and a low proportion of TME infiltrating immune cells. This phenotype might be the result of immunological ignorance, the induction of immune tolerance, and a lack of appropriate environment for T cell activation, and hence, reflected the absence of pre-existing anti-tumor immune response (Gajewski et al., 2013; Herbst et al., 2014; Hegde et al., 2016; Kim and Chen. 2016). Notably, we found several innate immune cells, such as eosinophil, mast cell, natural killer cell, and plasmacytoid dendritic cell, were enriched in the TME of pattern-A, while the patients in this pattern did not present a matching preferable prognosis as pattern B. Conceptually, the immune-excluded phenotype was characterized by stromal activation and the presence of vast immune cells, while they were retained in the stroma surrounding the tumor cells nests rather than penetrated the tumor parenchyma (Salmon et al., 2012; Herbst et al., 2014; Joyce and Fearon, 2015; Hegde et al., 2016). Previous study also provided evidence that T-cells proliferation and activation were observed after anti-PD1/PD-L1 agents but no infiltration (Herbst et al., 2014). Therefore, pattern-A was classified as immune-excluded phenotype, presenting a pre-existing anti-tumor immune response while being rendered ineffective by the retention of immune cells in the surrounding stroma. Generally, the immune-desert and -excluded phenotypes were both considered as the cold tumor, containing numerous immune-suppressive cytokines that contributed to impairing the anti-tumor response. These could explain the alike poorer prognosis of pattern-A and -C.

We next screened DEGs among three patterns, and GO functional annotation revealed they were significantly associated with DNA modification and damage repair related pathways, suggesting the different clinical and biological characteristics among three patterns might be the results of differentially expressed of these genes. We further identified prognostic DEGs, termed as DNA methylation signature genes, to perform unsupervised clustering. Likewise, we found three genomic subtypes, named DNA methylation gene clusters, whose clinical outcomes and immune cell infiltrating traits were similar to three modification patterns. Our comprehensive analyses strongly revealed three immune phenotypes in colon cancer with distinct clinical and TME immune characteristics, which enhanced our understanding of the non-negligible impact of DNA methylation in shaping different TME landscapes.

Whereas, above analyses were performed based on the patient population, which could not accurately predict the specific modification pattern in individual patients. As a consequence, based on the above signature genes, we developed the DNA methylation score, DMS, to quantify the certain modification pattern for each colon cancer patient. We found DMS could precisely discriminate three immune phenotypes, with the lowest median DMS in immune-inflamed type and the highest median DMS in immune-desert type. Additionally, we revealed a markedly positive relation between DMS and tumor stage, with DMS increasing gradually from the stage I to IV. Moreover, DMS was an independent prognostic risk factor, and patients with high DMS presented an inferior survival, which was validated in multiple colon cancer cohorts. Integrally, above results indicated DMS was a reliable tool to reflect the individual DNA methylation modification pattern, and predict clinical outcomes of colon cancer.

Colon cancer was a highly heterogeneous disease, resulting from a series of distinct genetic and epigenetic changes, and a subset of molecular alterations was considered to drive the cellular and clinical behavior of cancer, including MSI, CIMP, CIN, BRAF, and KRAS mutations (Marisa et al., 2013; Phipps et al., 2015). The CIMP subtype was referred to a distinct epigenome with a high frequency of methylated genes, and approximately 20% of colon cancer were CIMP+ tumors (Toyota et al., 1999). Previous study demonstrated that CIMP+ colon tumors had a unique association with BRAF^*V600E*^ oncogene mutation, and CIMP-associated methylation of MLH1 induced mismatch repair deficiency and resulted in a genomic instability status, also known as MSI, to generate more mutation burden and neoantigen (Weisenberger et al., 2006). In addition, aberrant epigenetic alterations as well contributed to the dysregulation of antigen-presenting machinery in tumor cells, leading to acquiring the adaptive immune response (Alexandrov et al., 2013). Here, we revealed DMS-low subtype was characterized as CIMP positive, mutant BRAF, MSI, and higher TMB, indicating this epigenotype of colon cancer identified by DMS had specific molecular alterations. Further analyses manifested the activation of immune and DNA damage repair related pathways, as well as abundant immune cells infiltration in the DMS-low group. Integrally, our findings substantiated DNA methylation modification was involved in the genomic instability, resulting in the accumulation of tumoral mutation burden and generation of neoantigen, which on the one hand activated the DNA damage repair pathway, and on the other hand enhanced immunogenicity to further activate the immune response.

Emerging evidence demonstrated that MSI and elevated TMB could heighten the anti-tumor activity of immunotherapy (Rizvi et al., 2015; Le et al., 2017; Mouw et al., 2017), and hence, we further investigated the ability of DMS to predict efficacy and prognosis of patients received anti-PD-1/PD-L1 antibody. In an immunotherapy cohort of advanced urothelial carcinoma patients treated with atezolizumab, we confirmed DMS-low group had a higher objective response rate and prolonged survival time than the DMS-high group. Higher PD-L1 expression and TMB value were considered to imply a favorable efficacy of immunotherapy (Gibney et al., 2016), and in this study, we discovered DMS was negatively correlated to TMB and PD-L1 expression in colon cancer, which validated the predictive value of DMS indirectly. Deeply, the pathway and immune phenotype analyses revealed activation of DNA damage repair and immune-inflamed environment in DMS-low patients, providing clues to illustrate the regulatory mechanism of DNA methylation in shaping TME immune landscape, and also confirmed patients with low DMS could benefit from immunotherapy. Collectively, we considered DMS had the potential to be an excellent predictive biomarker for immune checkpoint inhibitor, and might promote personalized colon cancer immunotherapy in the future.

A major limitation of this work was the public survival and transcriptomic data of colon cancer immunotherapy was not accessible yet. Therefore, the predictive performance of DMS needed to be further certified in the colon cancer immunotherapeutic cohort.

In conclusion, for the first time, we uncovered three distinct DNA methylation modification patterns in colon cancer, and illustrated their extensive regulatory mechanism in tumor immune environment formation, which was a non-negligible factor to cause individual TME heterogeneity and different clinical outcomes. Our integrated analyses of DNA methylation modification would contribute to enhancing the understanding of tumor immune characteristics, and providing novel insights to guide immunotherapy more effectively.

## Data Availability

All data analyzed in this study were available from The Cancer Genome Atlas (TCGA, https://portal.gdc.cancer.gov/), and Gene Expression Omnibus (GEO, https://www.ncbi.nlm.nih.gov/geo/).
