# Supplementary material for: DNA methylation regulator-mediated modification pattern defines tumor microenvironment immune infiltration landscape in colon cancer
Source: Front Genet. 2022 Oct 6;13:1008644. doi: 10.3389/fgene.2022.1008644 (PMC9582351; doi:10.3389/fgene.2022.1008644)
Supplement: Supplementary file 1 [file DataSheet1.docx]

**Supplementary Figures**

**
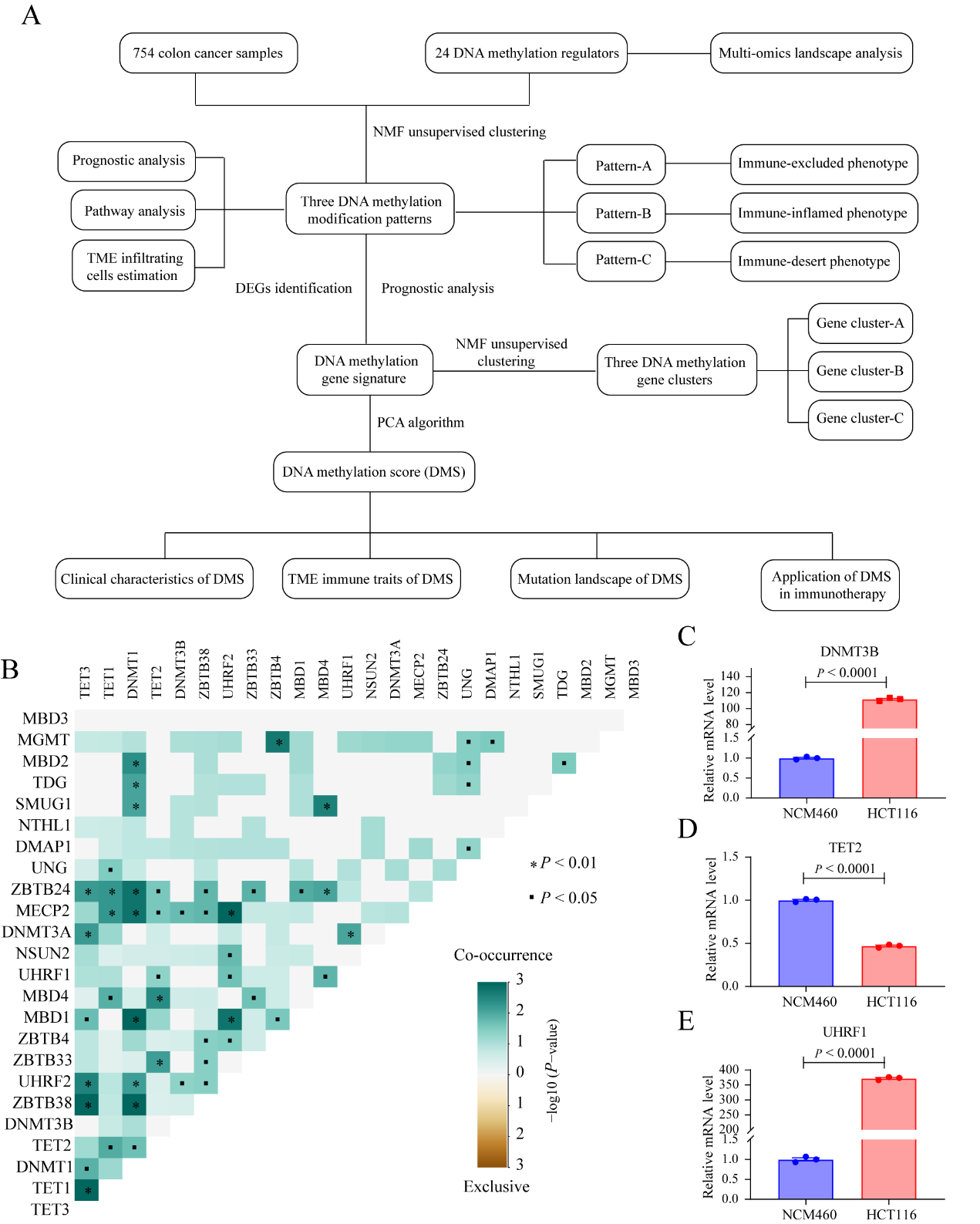
**

**Figure S1. Workflow of study and mutation analysis of 24 DNA methylation regulators**

**A).** Workflow of this study.

**B).** The mutation co-occurrence and exclusive analyses of 24 DNA methylation regulators.

**C-E).** The qRT-PCR determined that the mRNA level of DNMT3B C) and UHRF1 E) were significantly higher in HCT116 cells while the mRNA level of TET2 D) was significantly higher in NCM460 cells.


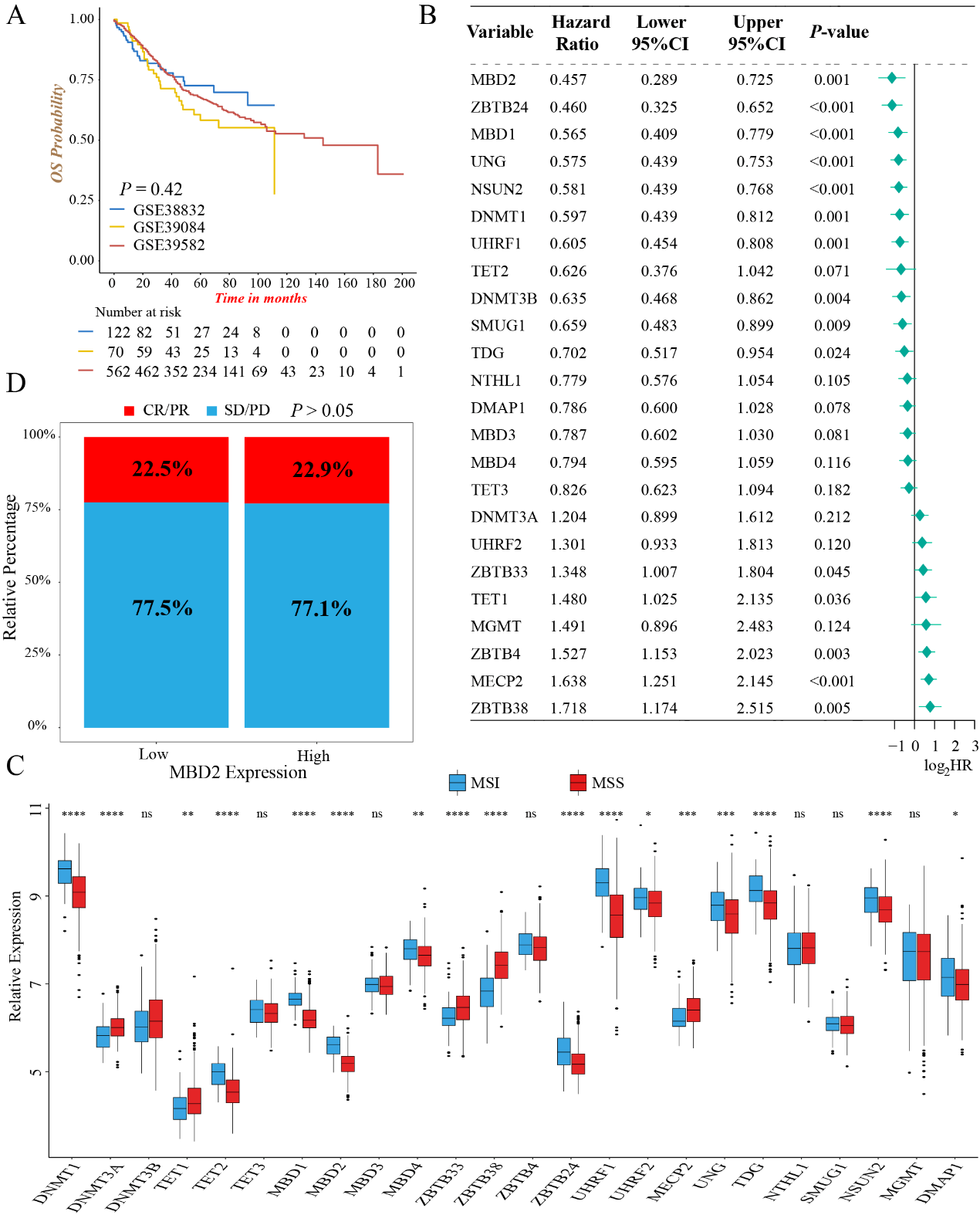


**Figure S2. Prognosis and molecular characteristics of 24 DNA methylation regulators**

**A).** Survival analysis among three GEO datasets (Log-rank test, *P* = 0.42).

**B).** The forest plot of the univariate Cox regression model revealed the prognostic value of 24 DNA methylation regulators in meta-cohort.

**C).** The relative expression of 24 DNA methylation regulators between the MSI and MSS groups (Wilcoxon test; ns: no significance; *: *P* < 0.05; **: *P* < 0.01; ***: *P* < 0.001; ****: *P* < 0.0001).

**D).** The proportion of patients with response to immunotherapy between the MBD2 high and low expression groups (Chi-square test, *P* > 0.05).


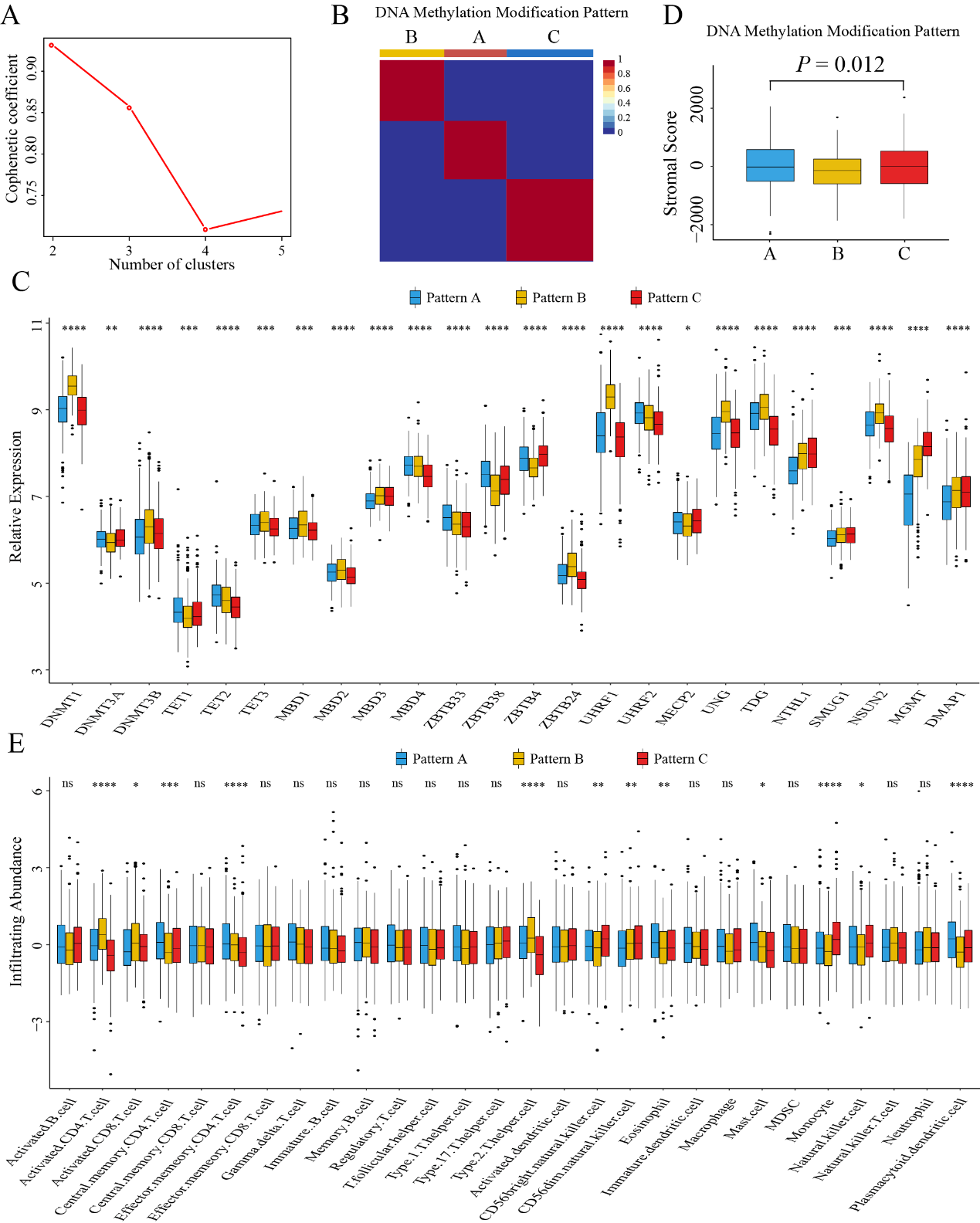


**Figure S3. DNA methylation modification patterns and their immune characteristics**

**A).** Cophenetic correlation coefficient. The k = 3 was the optimal k value.

**B).** Consensus matrix.

**C).** The relative expression of 24 DNA methylation regulators among three DNA methylation modification patterns in meta-cohort (Kruskal-Wallis test; *: *P* < 0.05; **: *P* < 0.01; ***: *P* < 0.001; ****: *P* < 0.0001).

**D).** Comparison of stromal score among three DNA methylation modification patterns (Kruskal-Wallis test; *P* = 0.012).

**E).** The infiltrating abundance of 28 TME cell types among three DNA methylation modification patterns (Kruskal-Wallis test; ns: no significance; *: *P* < 0.05; **: *P* < 0.01; ***: *P* < 0.001; ****: *P* < 0.0001).


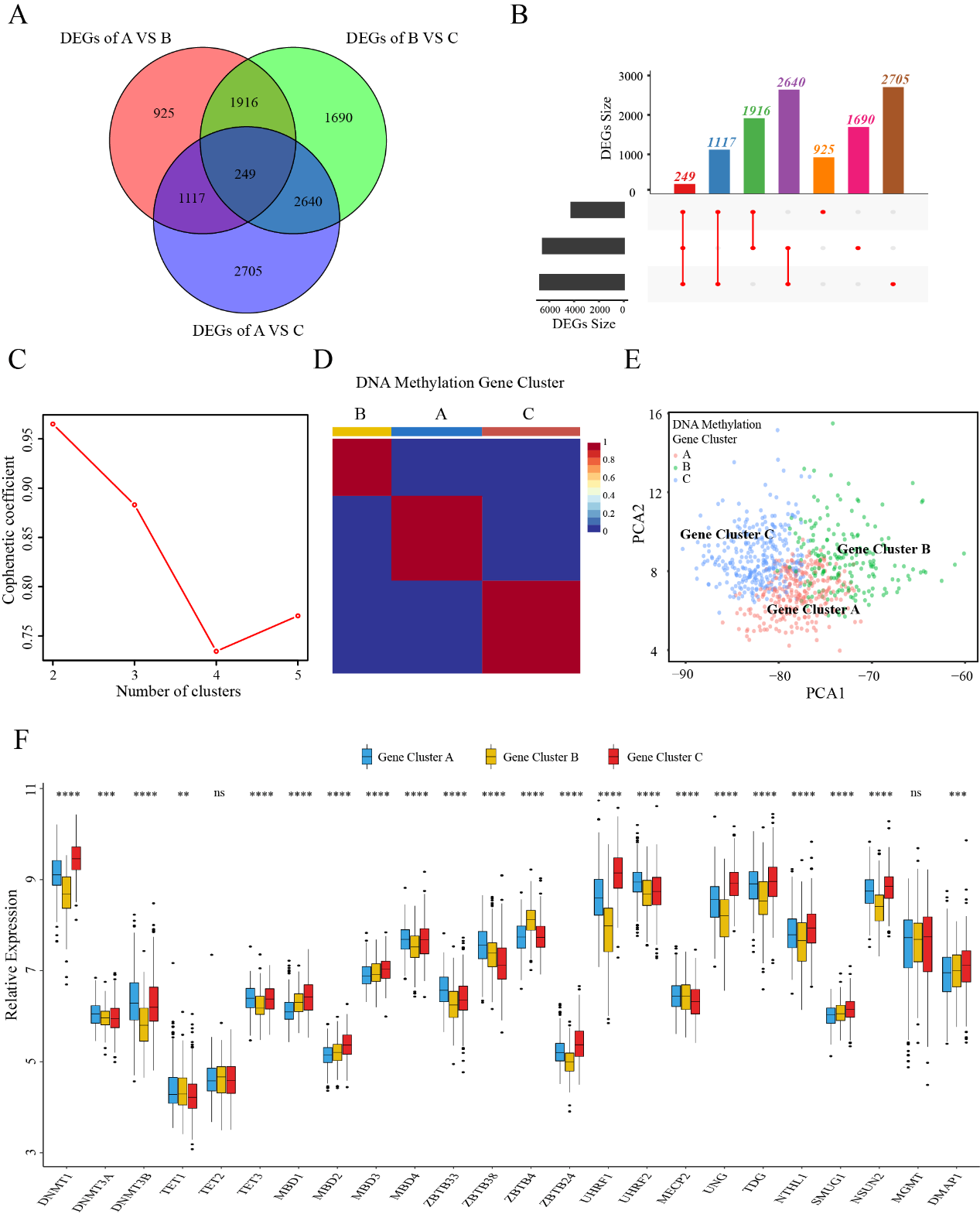


**Figure S4. Generation of DNA methylation gene signature**

**A-B).** Venn diagram **A)** and Upset plot **B)** showed 249 DEGs among three DNA methylation modification patterns.

**C).** Cophenetic correlation coefficient. The k = 3 was the optimal k value.

**D).** Consensus matrix

**E).** PCA graph for the transcriptomic profiles of three DNA methylation gene clusters, showing a remarkable difference among three gene clusters.

**F).** The relative expression of 24 DNA methylation regulators among three DNA methylation gene clusters in meta-cohort. (Kruskal-Wallis test; ns: no significance; *: *P* < 0.05; **: *P* < 0.01; ***: *P* < 0.001; ****: *P* < 0.0001).


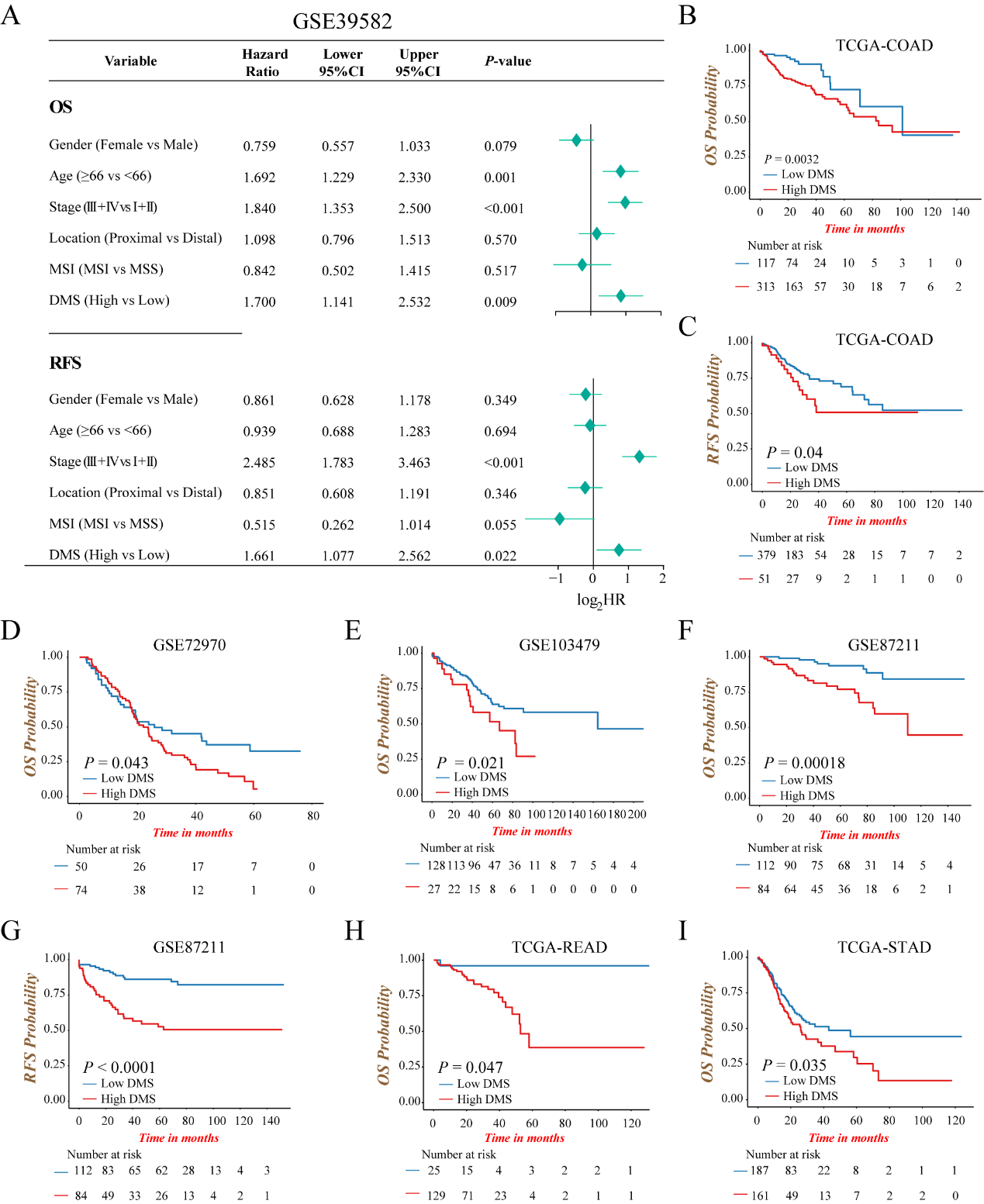


**Figure S5. Prognostic value of DNA methylation score in multiple colon cancer cohorts**

**A).** Multivariate Cox regression analysis of OS and RFS for DMS in the GSE39582 cohort shown by the forest plot.

**B).** Overall survival analysis for low and high DMS groups in the TCGA-COAD cohort (Log-rank test, *P* = 0.0032).

**C).** Recurrence-free survival analysis for low and high DMS groups in the TCGA-COAD cohort (Log-rank test, *P* = 0.04).

**D).** Overall survival analysis for low and high DMS groups in the GSE72970 cohort (Log-rank test, *P* = 0.043).

**E).** Overall survival analysis for low and high DMS groups in the GSE103479 cohort (Log-rank test, *P* = 0.021).

**F).** Overall survival analysis for low and high DMS groups in the GSE87211 cohort (Log-rank test, *P* = 0.00018).

**G).** Recurrence-free survival analysis for low and high DMS groups in the GSE87211 cohort (Log-rank test, *P* < 0.0001).

**H).** Overall survival analysis for low and high DMS groups in the TCGA-READ cohort (Log-rank test, *P* = 0.047).

**I).** Overall survival analysis for low and high DMS groups in the TCGA-STAD cohort (Log-rank test, *P* = 0.035).


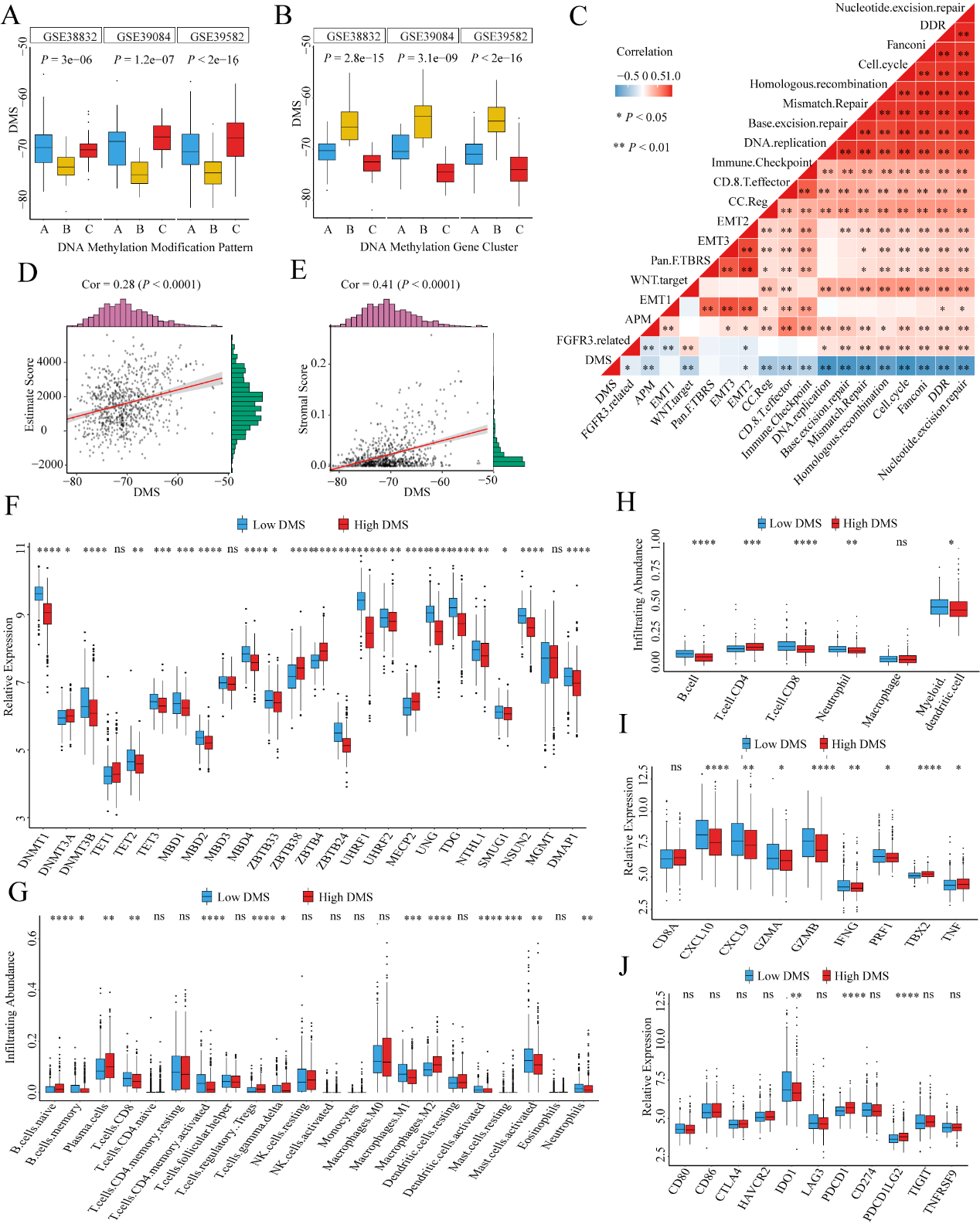


**Figure S6. Characterization of DMS in TME and biological processes**

**A-B).** Differences in DMS among three DNA methylation modification patterns **A)** and three DNA methylation gene clusters **B)** in three separate GSE cohorts (Kruskal-Wallis test, all *P* < 0.001).

**C).** Correlations between DMS and the known biological processes through Spearman analysis (Spearman correlation analysis, *: *P* < 0.05; **: *P* < 0.01).

**D).** The scatter plot depicted the positive correlation between DMS and estimate score (Spearman correlation analysis, *P* < 0.0001).

**E).** The scatter plot depicted the positive correlation between DMS and stromal score (Spearman correlation analysis, *P* < 0.0001).

**F).** The relative expression of 24 DNA methylation regulators between DMS-high and -low groups (Wilcoxon test; ns: no significance; *: *P* < 0.05; **: *P* < 0.01; ***: *P* < 0.001; ****: *P* < 0.0001).

**G)** The CIBERSORT method identified infiltrating abundance of different TME immune cells between DMS-high and -low groups (Wilcoxon test; ns: no significance; *: *P* < 0.05; **: *P* < 0.01; ***: *P* < 0.001; ****: *P* < 0.0001).

**H)** The TIMER database identified infiltrating abundance of different TME immune cells between DMS-high and -low groups from TCGA-COAD cohort (Wilcoxon test; ns: no significance; *: *P* < 0.05; **: *P* < 0.01; ***: *P* < 0.001; ****: *P* < 0.0001).

**I).** The difference in the immune activation-related gene expression between DMS-high and -low groups (Wilcoxon test; ns: no significance; *: *P* < 0.05; **: *P* < 0.01; ***: *P* < 0.001; ****: *P* < 0.0001).

**J).** The difference in the immune checkpoint-related gene expression between DMS-high and -low groups (Wilcoxon test; ns: no significance; *: *P* < 0.05; **: *P* < 0.01; ***: *P* < 0.001; ****: *P* < 0.0001).


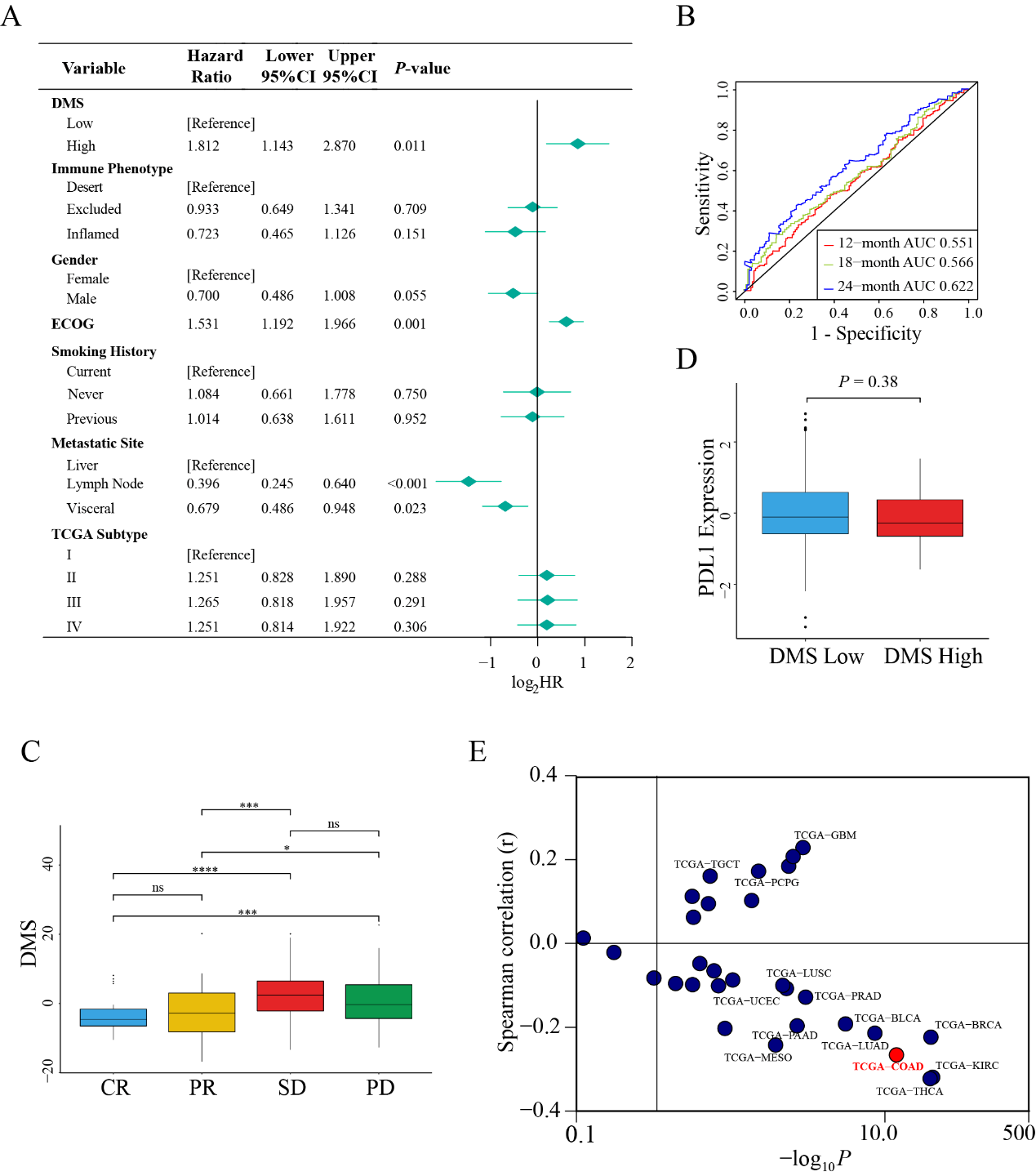


**Figure S7. The predictive value of DMS in immunotherapy**

**A).** Multivariate Cox regression analysis of OS for DMS in the IMvigor210 cohort shown by the forest plot.

**B).** ROC curves to reflect the ability of DMS to predict the 12-, 18-, 24-months survival probability for patients received immunotherapy in the IMvigor210 cohort.

**C).** Comparison of DMS among different responsive groups for immunotherapy in the IMvigor210 cohort (Kruskal-Wallis test; ns: no significance; *: *P* < 0.05; **: *P* < 0.01; ***: *P* < 0.001; ****: *P* < 0.0001). SD, stable disease; PD, progressive disease; CR, complete response; PR, partial response.

**D).** Comparison of PD-L1 expression between DMS-high and -low groups in the IMvigor210 cohort (Wilcoxon test; *P* = 0.38).

**E).** Correlations between DMS and PD-L2 expression in pan-cancer cohorts through Spearman analysis.
